# Cultivar-specific dynamics: unravelling rhizosphere microbiome responses to water deficit stress in potato cultivars

**DOI:** 10.1186/s12866-023-03120-4

**Published:** 2023-12-01

**Authors:** Benoit Renaud Martins, Roberto Siani, Krzysztof Treder, Dorota Michałowska, Viviane Radl, Karin Pritsch, Michael Schloter

**Affiliations:** 1 Research Unit for Comparative Microbiome Analysis (COMI), Helmholtz Zentrum München, German Research Center for Environmental Health (GmbH), Neuherberg, Germany; 2 Chair for Environmental Microbiology, Technical University of Munich, Freising, Germany; 3 Plant Breeding and Acclimatization Institute – National Research Institute in Radzików, Bonin Division, Department of Potato Protection and Seed Science at Bonin, 76-009, Bonin, Bonin Str 3 Poland; 4 Research Unit for Environmental Simulation (EUS), Helmholtz Zentrum München, German Research Center for Environmental Health (GmbH), Neuherberg, Germany

**Keywords:** Potato rhizosphere, Soil microbiome, Drought tolerance, Actinobacteria, Rhizobiales

## Abstract

**Background:**

Growing evidence suggests that soil microbes can improve plant fitness under drought. However, in potato, the world’s most important non-cereal crop, the role of the rhizosphere microbiome under drought has been poorly studied. Using a cultivation independent metabarcoding approach, we examined the rhizosphere microbiome of two potato cultivars with different drought tolerance as a function of water regime (continuous versus reduced watering) and manipulation of soil microbial diversity (i.e., natural (NSM), vs. disturbed (DSM) soil microbiome).

**Results:**

Water regime and soil pre-treatment showed a significant interaction with bacterial community composition of the sensitive (HERBST) but not the resistant cultivar (MONI). Overall, MONI had a moderate response to the treatments and its rhizosphere selected Rhizobiales under reduced watering in NSM soil, whereas *Bradyrhizobium*, *Ammoniphilus*, *Symbiobacterium* and unclassified Hydrogenedensaceae in DSM soil. In contrast, HERBST response to the treatments was more pronounced. Notably, in NSM soil treated with reduced watering, the root endophytic fungus *Falciphora* and many Actinobacteriota members (*Streptomyces*, *Glycomyces*, *Marmoricola*, *Aeromicrobium*, *Mycobacterium* and others) were largely represented. However, DSM soil treatment resulted in no fungal taxa and fewer enrichment of these Actinobacteriota under reduced watering. Moreover, the number of bacterial core amplicon sequence variants (core ASVs) was more consistent in MONI regardless of soil pre-treatment and water regimes as opposed to HERBST, in which a marked reduction of core ASVs was observed in DSM soil.

**Conclusions:**

Besides the influence of soil conditions, our results indicate a strong cultivar-dependent relationship between the rhizosphere microbiome of potato cultivars and their capacity to respond to perturbations such as reduced soil moisture. Our study highlights the importance of integrating soil conditions and plant genetic variability as key factors in future breeding programs aiming to develop drought resistance in a major food crop like potato. Elucidating the molecular mechanisms how plants recruit microbes from soil which help to mitigate plant stress and to identify key microbial taxa, which harbour the respective traits might therefore be an important topic for future research.

**Supplementary Information:**

The online version contains supplementary material available at 10.1186/s12866-023-03120-4.

## Introduction

Ongoing global warming leads to rapid changes in precipitation patterns and temperature, resulting in increased drought stress in many regions worldwide, which poses challenges for crop productivity and subsequently global food security [1, 2], as it induces numerous adverse effects on plants including decreased growth, biomass, photosynthetic activities and an increase in oxidative damages [3–5]. Thus, drought tolerance is one of the major aims in breeding programs for crops [6]. Potato is the most produced vegetable crop worldwide [7], and among the hundreds of commercial cultivars, there is a considerable variation in drought tolerance [8–10]. Since very short periods of water shortage can lead to substantial yield loss [11], potato is generally regarded as drought-sensitive crop [12]. Drought susceptibility has been mainly attributed to its shallow root system [13, 14] with weak soil penetration [15, 16], but there are also complex environmental interactions [17]. The development of potato roots is regulated by auxin signaling pathways, however, in addition, diverse factors including early tuber initiation, elevated nitrate concentrations, and plant age collectively suppress further root growth, ultimately leading to the reduced root length density observed in potatoes [15]. Thus, besides breeding efforts in potato [18], additional approaches particularly the use of plant growth promoting and fortifying microbes are considered an important component to improve plant health and performance [19, 20].

Positive effects of soil microbial diversity have been associated with soil multifunctionality (nutrient cycling, primary production, litter decomposition etc.) [21]. Particularly, microbes inhabiting the plant rhizosphere and their complex interactions with the host plant significantly affect plant morphology, physiology, growth, development, and health [22]. Therefore, plant-microbe interactions are thought to play a critical role in the fast adaptation of plants to environmental stress conditions [23].

Considered as the second plant genome [24–26], the rhizosphere microbiome contributes to a broad spectrum of functions including plant nutrition, defense against plant pathogens, and adaptation to abiotic and biotic stresses [27, 28]. Rhizosphere microbial populations are influenced by a complex combination of factors. Among these, soil acting as microbial seed bank plays the most significant role in determining the composition of rhizosphere microbial communities as shown for populus [29], cotton [26] and soybean [30], as well as for potato with cultivars grown in two different soils recruiting different microbial communities [31].

The rhizosphere microbiome can also be affected by environmental factors including drought. Drought-induced transformations in the microbiome have been attributed to changes in plant physiology and biochemistry, which compromise the quantity of carbon compound efflux and root exudate compositional profiles [32–34]. Moreover, varieties (cultivars) of a number of plant families with different levels of drought tolerance showed distinct responses to drought in their respective rhizosphere microbiomes as evidenced in alfalfa [35], sugarcane [36], broomcorn millet [37], rice [38] and tomato [39]. These differences include the recruitment of Proteobacteria or Firmicutes members in drought tolerant and Actinobacteria in drought sensitive cultivars [36, 37]. Another study involving 18 grass species demonstrated that Actinobacteria enrichment was more pronounced as the plant-microbe interactions increased (endosphere > rhizosphere > surrounding soil) [40], implying that they are important for plant response to drought. This was further exemplified in an inoculation assay, in which the root colonization of the Actinobacteria genus *Streptomyces* was consistent with increased root growth of *Sorghum bicolor* under drought [41]. These results underline that specific microbial taxa are associated with plant growth under drought.

Diverse mechanisms behind microbial-mediated drought resistance in plants have been previously reported, notably those modulating phytohormone levels in stressed plants [42]. This includes mechanisms reducing levels of the plant stress hormone ethylene by amino-1-cyclopropane carboxylate (ACC) deaminase-producing bacteria [43], particularly in drought-enriched Actinobacteria [44]. The microbial production of auxins has also been suggested to improve root traits related to drought tolerance such as root length, number of root tips and surface area [45].

There is no clear pattern on how drought affects fungal communities in soil and plant rhizosphere. While many studies showed no or minor effects of drought on fungal community composition [40, 46–48], other studies found significant changes under drought [38, 49, 50].

In potato, little is known on how drought affects the interactions between potato and its rhizosphere microbiomes. The aim of this study was to investigate the potential role of the rhizosphere microbiome in sustaining potato growth under reduced soil moisture. We introduced in our system, plant genetic variability by using two potato cultivars of contrasting drought tolerance. Additionally, plants were cultivated in diverse soil conditions consisting of a soil with a natural microbiome and another with an artificially reduced microbiome. This factor was included in the system given that the rhizosphere microbiome is significantly influenced by the soil microbial background. We compared the rhizosphere microbiome (bacteria, fungi) of each cultivar between continuous and reduced watering. Given that plants interact with a myriad of microorganisms at the roots, resulting in unique rhizosphere microbial associations that respond to environmental conditions [23], we hypothesized that when drought tolerance is mediated in potato by the recruitment of beneficial microbes, the rhizosphere microbiome would be affected in a cultivar-specific manner under reduced watering, with the resistant cultivar exhibiting a more drought adaptive microbiome than the sensitive cultivar (**H1**).

For the soil with an artificially reduced microbiome, we hypothesized that when the rhizosphere microbiome plays a dominant role in drought tolerance of the two potato cultivars, then a reduction of the soil microbiome would result in lower drought tolerance under reduced watering in both cultivars (**H2**).

## Materials and methods

### Soil sampling and analysis of physico-chemical properties

In spring 2020, top of luvisol (0–20 cm) characterised as a silty loam was obtained from the Gut-Roggenstein experimental station (latitude 48.1879670, longitude 11.3342012, 508 m above sea level), Technical University of Munich in Southern Germany. The soil contained 1.27% total carbon and 0.1% total nitrogen resulting in a C:N ratio of 12.7. In previous years, the site underwent consecutive crop rotations such as summer barley in 2015, sugar beet in 2016, rapeseed in 2017, wheat in 2018, winter barley in 2019 and sugar beet in 2020. The soil was sieved with a 2-mm diameter mesh and divided into two portions. One was autoclaved for 20 min at 121 °C (to reduce soil microbial biomass and diversity) and the other was left in its native state. Before potting and planting, both soils were stored at 8 °C for 7 days.

Bare NSM and DSM soils (source soils) were characterised for microbial biomass carbon (Cmic) and nitrogen (Nmic), using chloroform fumigation-based extraction method [51, 52], which were calculated as the difference between total dissolved organic carbon (DOC) and nitrogen (DON) in fumigated and non-fumigated samples, with an extraction efficiency coefficient (k_ec_) value of 0.45 for carbon [52] and k_en_ value of 0.54 for nitrogen [53]. Inorganic nitrogen (NH_4_^+^ and NO_3_^−^), pH, soil texture (clay, silt and sand contents), magnesium (Mg), phosphorus (P_2_O_5_) and potassium (K_2_O) as well as the maximum water holding capacity (mWHC) were also determined using standard protocols. Details on protocols used and soil characteristics are summarised in the supplementary document (Table S1, S2).

### Experimental design

**Fig. 1:**
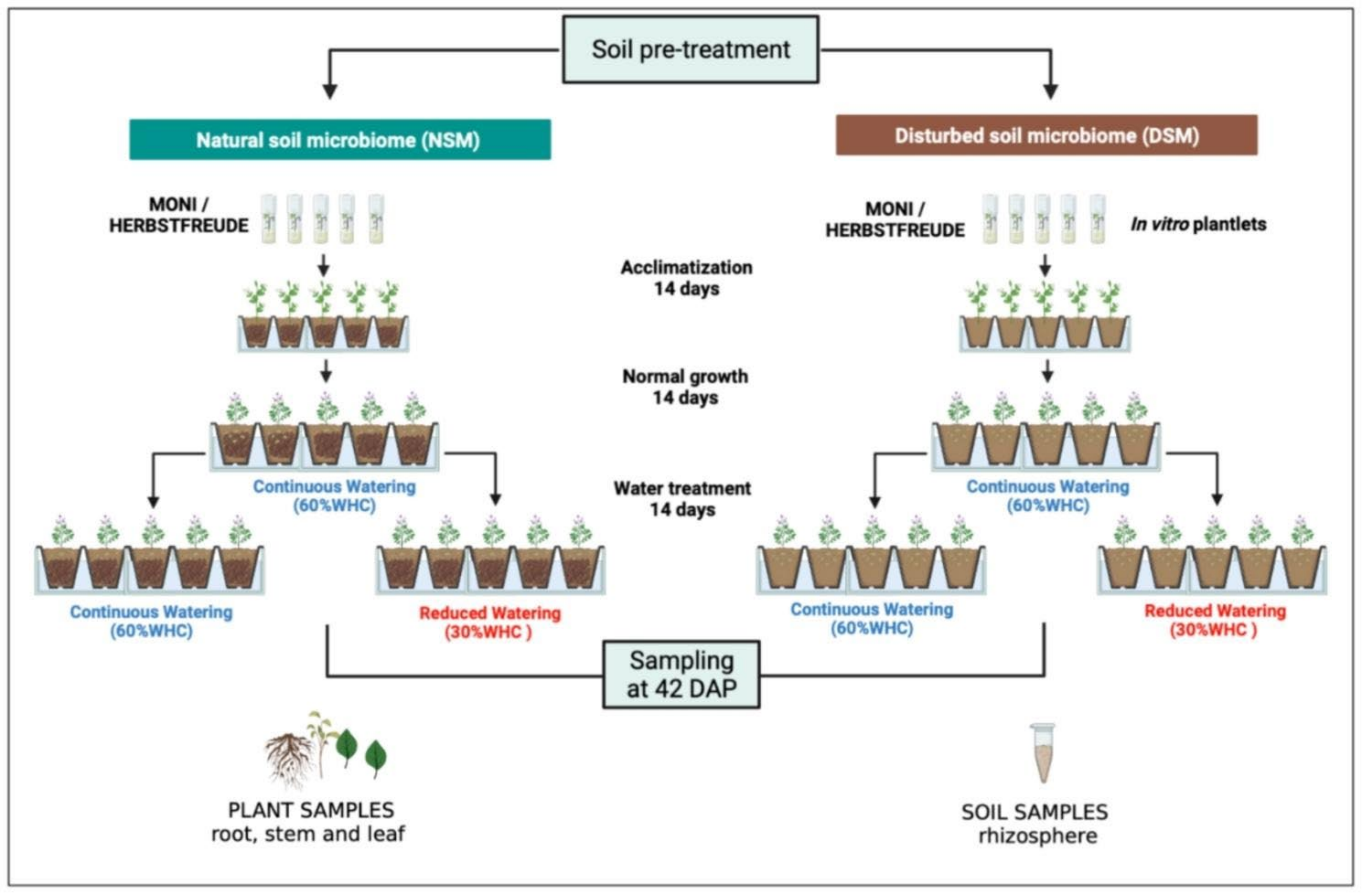
Experimental design of the greenhouse experiment using two in vitro propagated potato cultivars (MONI, HERBST) acclimated in native soil with natural microbiome (NSM) and autoclaved soil with disturbed microbiome (DSM) for 14 days. After transfer to 1.5 L pots containing the corresponding soils, plants were grown for another 14 days under continuous watering (60% maximum water holding capacity (mWHC)). After 28 days, plants grown either in NSM or DSM soils were divided in two groups, one was constantly kept under continuous watering whereas the second was kept under reduced watering (RW = 30% mWHC). 14 days after imposition of water regimes, root, stem, leaf samples were taken to assess growth traits and rhizosphere soil samples were collected for bacterial and fungal community analyses. Created with BioRender.com

Two potato (*Solanum tuberosum*) cultivars were used in this experiment (Fig. 1), MONI referenced as drought-resistant and HERBSTRFREUDE (HERBST used throughout the text) considered as drought-sensitive (personal observation, K. Treder).

Additional information about the two cultivars is available on the European cultivated potato database [54]. Both cultivars were vegetatively propagated as tissue cultures at the Institute of Plant Breeding and Acclimation in Bonin (Bonin, Poland). Plant tissues were grown in test tubes under in vitro conditions using Murashige and Skoog nutrient medium for approximately 8 weeks. Certified healthy plants (pathogen-free) were delivered to Helmholtz Munich (Neuherberg, Bavaria, Germany). Agar plugs attached to the roots of the plantlets were gently removed with tweezers and tap water. Plantlets were immediately transferred to 0.3 L (7 × 7 × 8 cm) pots filled with NSM or DSM soils and allowed to acclimate for 2 weeks. During the acclimation period, the early-stage plants were watered thrice per week.

The acclimated plants were afterwards transferred to 1.5 L (11 × 11 × 12 cm) pots, in which they were grown in a greenhouse for 14 days under optimal conditions (RH: 65%; day/night temperature: 22 °C/18 °C day/night natural photoperiod: 16/8) and maintained with continuous watering (CW = 60% mWHC). Subsequently, plants grown either in NSM or DSM soils were divided in two groups, one was continuously kept under continuous watering whereas the second was kept with reduced watering (RW = 30% mWHC). The entire trial was conducted without applying fertilisers to not influence the soil microbiome. Three unplanted pots each filled with NSM and DSM soils served as controls for water content adjustments throughout the experiment. 14 days after plants were imposed to the two water regimes the experiment was completed. Plant growth parameters including stem height, leaf dry mass, root length and fresh weight were measured. The soil adhering to the plant roots, defined as the rhizosphere, was collected per plant. Based on 2 soil pre-treatments x 2 water regimes x 2 cultivars x 5 replicates, the experiment yielded 40 rhizosphere samples for analyses. After autoclaving and prior to planting, NSM and DSM source soils were sampled for assessment of the initial soil microbiome (T0). Source soils and rhizosphere samples were stored at -80 °C until DNA extraction.

### DNA extraction and library preparation for amplicon sequencing

DNA was extracted from 400 mg of soil samples taken at T0 and from the rhizosphere at day 42, according to [55]. Empty extraction tubes were used as negative controls to check for contamination during the process. The concentration of total DNA extracts was quantified in duplicate using SpectraMax Gemini EM Microplate Spectrofluorometer (Molecular Devices, CA, USA) and Quant-iT PicoGreen dsDNA assay kit (Thermo Fischer Scientific, Waltham, USA) according to the manufacturer’s instructions. All samples were stored at -20 °C until further analysis.

The ITSmix3/ITSmix4 primer pair [56], was used to amplify the ITS2 region of the fungal nuclear rDNA. PCR was performed with an initial denaturation phase at 95 °C for 15 min and 30 cycles of 30 s denaturation at 95 °C, 30 s annealing at 55 °C and 1 min extension at 72 °C and a final extension of 10 min at 72 °C. PCR reaction mixtures contained 2 µl of 10 ng DNA templates, 0.2 µl of 10 pmol of each primer, 2.5 µl of 3% BSA, 12.5 µl of NEBNext High-Fidelity 2x PCR Master Mix (New England Biolabs, Frankfurt am Main, Germany) and 7.6 µl of DEPC-treated water, resulting in a total volume of 25 µl.

Amplification of the V4 region of the bacterial 16S rRNA gene required the use of universal primer pair 515F/806R [57, 58]. PCR was performed under the following conditions: an initial denaturation phase at 98 °C for 1 min and 30 cycles of 10 s denaturation at 98 °C, 30 s annealing at 55 °C and 30 s extension at 72 °C and a final extension for 5 min at 72 °C. PCR reaction mixtures contained 2.6 µl of 10 ng DNA templates, 0.2 µl of 10 pmol of each primer, 2.5 µl of 3% BSA, 12.5 µl of NEBNext High-Fidelity 2x PCR Master Mix (New England Biolabs, Frankfurt am Main, Germany) and 7 µl of DEPC-treated water.

PCR products were verified in 1% agarose gels, followed by MagSi NGSprep Plus bead purification (Steinbrenner, Wiesenbach, Germany). The quality and quantity of purified amplicons and the presence of primer dimers were checked with DNF-473 Standard Sensitivity NGS Fragment Kit (1-6000 bp) on a fragment analyser (Agilent Technology, Santa Clara, California, USA). Amplicons were diluted to 2ng/µl. 8-cycle indexing PCR was performed in a reaction mixture (25 µl) using 2 µl of each indexing primer (Nextera® XT Index Kit v2; Illumina, San Diego, California, United States), 12.5 µl NEBNext High-Fidelity 2x PCR Master Mix, 1.5 µl DEPC-treated water and 5 µl of 10 ng purified amplicon. The indexed amplicons underwent a second round of purification, followed by quality and quantity control as described above. Prior to sequencing, samples were normalised to 4 nM and equimolarly pooled into a single Eppendorf tube. Paired-end sequencing was carried out using the MiSeq ® Reagent kit v3 (600 cycles) (Illumina Inc., San Diego, California, USA).

### Pre-processing of sequencing data

Raw sequencing data were analysed using the Galaxy web platform [59]. After the raw data were imported into the platform, forward and reverse FASTQ files were used to build their respective dataset lists. Forward and reverse dataset lists were trimmed with a minimum read length of 50 using the Cutadapt function [60]. Quality control for the forward and reverse reads was performed via FastQC [61]. Subsequently, data analysis was performed using the DADA2 pipeline (Galaxy Version 1.20) [62]. The following trimming and filtering parameters were considered for 16S rRNA analysis: 20 bp were removed n-terminally and reads were truncated at position 230 (forward) and 180 (reverse) with expected error of 3 and 4, respectively. For the ITS2 region analysis, forward reads were trimmed to 20–220 bp, reverse reads to 20–160 bp and same number of expected errors was used. After merging reads, the resulting unique amplicon sequence variants (ASVs) were used for taxonomic assignments. ASVs are biological sequences discriminated from errors, allowing the detection of single-nucleotide differences over the sequenced genes.

For bacterial taxonomic assignment, ASVs were trained against SILVA database v138.1 with 0.99 confidence threshold. Fungal ASVs were taxonomically assigned using UNITE fungi database v9.0 released for QIIME with 0.99 confidence threshold [63]. Amplicon sequences from bacteria and fungi were aligned and phylogenetic trees were constructed. The R language and environment v4.2.1 were used for downstream analysis. Using Bioconductor decontam package v1.13.0 [64], contaminant sequences were filtered leveraging the negative controls, along with ASVs assigned to chloroplast and mitochondria. A phyloseq object was created for each of bacterial and fungal datasets using the Phyloseq package v1.42.0. Singletons (ASVs represented by only one read across all samples) were removed. Furthermore, only ASVs consistently found in 80% of the biological replicates (4 out of 5) in each sample collection were kept for downstream analysis. We employed Total-Sum Scaling (TSS) for data normalization. TSS involves transforming the abundance table into a relative abundance table by scaling the data according to the library size of each sample.

### Statistical analyses

Bare NSM and DSM soils were characterised regarding physico-chemical properties and statistical differences were calculated either with a paired Student t-test or Wilcoxon test. Shapiro-Wilk test was used to assess the normality of the distribution in each group prior to conducting the t-test. *p*-values greater than the significance level of 0.05 indicate that the distribution is not significantly different from a normal distribution.

Statistical differences between plant growth parameters in soil pre-treatment were calculated using the non-parametric Wilcoxon test. Throughout the text, the significance level for all statistical tests was set at 0.05.

For α-diversity estimation of bacterial and fungal communities in the plant rhizosphere, observed species and Shannon index were calculated across sample groups and visualized using the packages Microbiome v1.20.0 and ggplot2 v3.4.0, respectively. Wilcoxon test was conducted to assess the effect of soil pre-treatment and water regimes on the α-diversity. To partition the source of variance, the relative contribution of soil pre-treatment, and water regimes was assessed using a permutational multivariate analysis of variance (PERMANOVA) based on UniFrac dissimilarity matrices as implemented in the adonis2 function (R package vegan v2.6-4).

The analysis of differentially abundant taxa across sample groups was conducted with LEfSe as implemented in the diff_analysis function (Bioconductor R package MicrobiotaProcess, v1.10.2) under one-against-all mode (i.e., one taxon is significantly different only when it is significantly different against all remaining treatments). In brief, a Kruskal-Wallis test, followed by a Wilcoxon test were used to isolate differentially abundant features. For overall abundance comparison between continuous and reduced watering across all microbial taxa, logarithmic LDA score threshold set to 2.5 was calculated and any taxa with α less than 0.05 were defined to be significantly different between water regimes. To provide a comprehensive comparison of microbial abundance across sample groups, the LEfSe analysis between continuous and reduced watering samples was performed at genus level.

Comparative Venn diagram analysis was performed to identify overlapping ASVs between the two cultivars amongst soil pre-treatment and water regimes. The shared microbiomes under the different treatments were defined using ASVs that were found in 80% of the replicates in each sample group with a relative abundance threshold of 0.001. The analysis was computed with the function amp_venn of the package ampvis2 (v2.7.34). The function amp_heatmap from the same package was used to visualise the shared microbiome composition and their relative abundances.

## Results

### Plant growth parameters

**Fig. 2:**
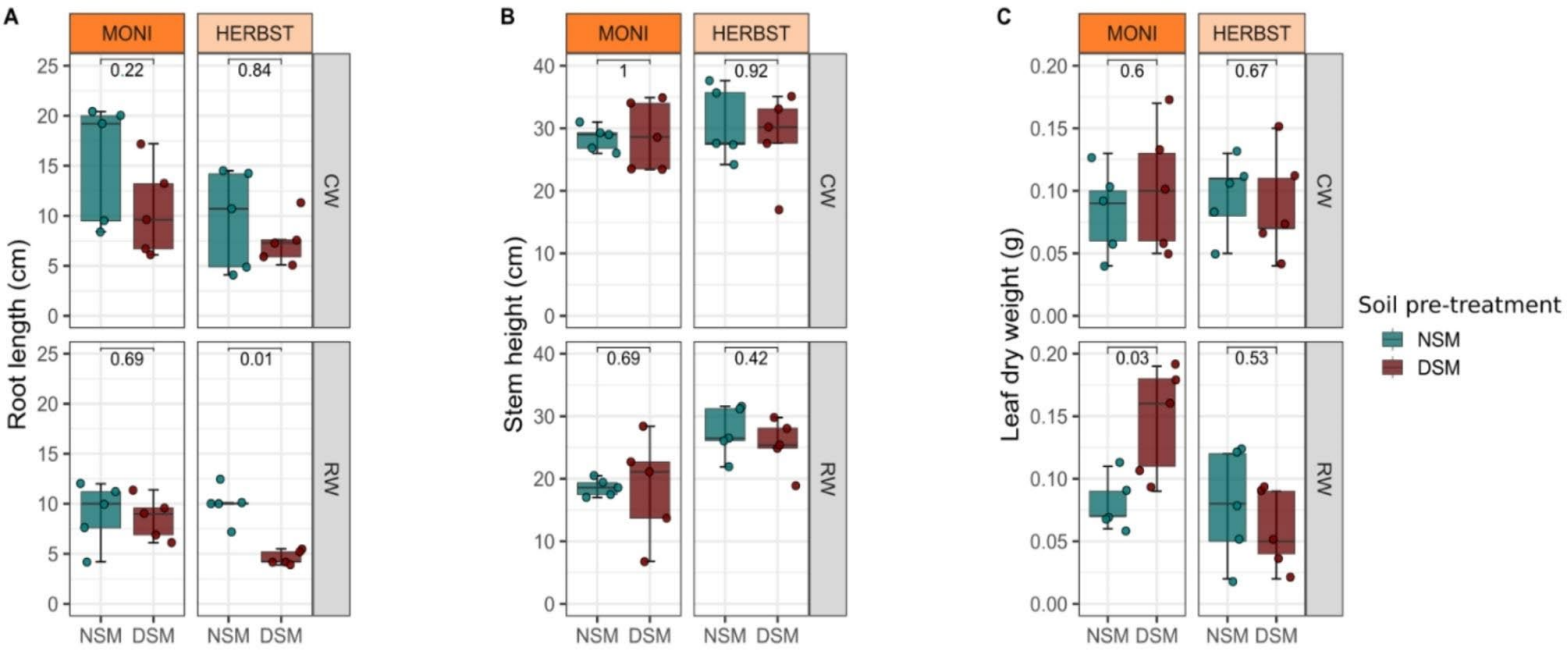
Plant growth parameters. (**A**) root length (**B**) stem height and (**C**) leaf dry weight of two potato cultivars MONI and HERBST were compared between native soil with natural microbiome (NSM, turquoise green) and autoclaved soil with disturbed microbiome (DSM, brown) under continuous watering (CW) and reduced watering (RW). Horizontal bars within boxes are the median. The tops and bottoms of the boxes represent 75th and 25th quartiles, respectively. The two vertical lines outside the boxes represent the whiskers. The colored dots stand for the individual observations. A non-parametric Wilcoxon test (*p* < 0.05, n = 5) was applied to calculate significant differences across sample groups and numbers above the boxes indicate the statistical *p*-values

Plant growth parameters revealed that soil pre-treatment and water regimes had no significant effect on root length of the cultivar MONI, whereas HERBST exhibited a significant reduction of its root length and root fresh weight when grown in DSM soil under reduced watering (Fig. 2A and Supplementary Figure S1). Aboveground, both treatments did not affect stem height in either cultivar (Fig. 2B). Surprisingly, MONI cultivated with reduced watering in DSM soil showed a significantly higher leaf dry weight compared to NSM soil (Fig. 2C) but no change in leaf dry weight of HERBST was observed.

### Effect of soil pre-treatment and water regimes on diversity and composition of rhizosphere microbiomes

**Fig. 3:**
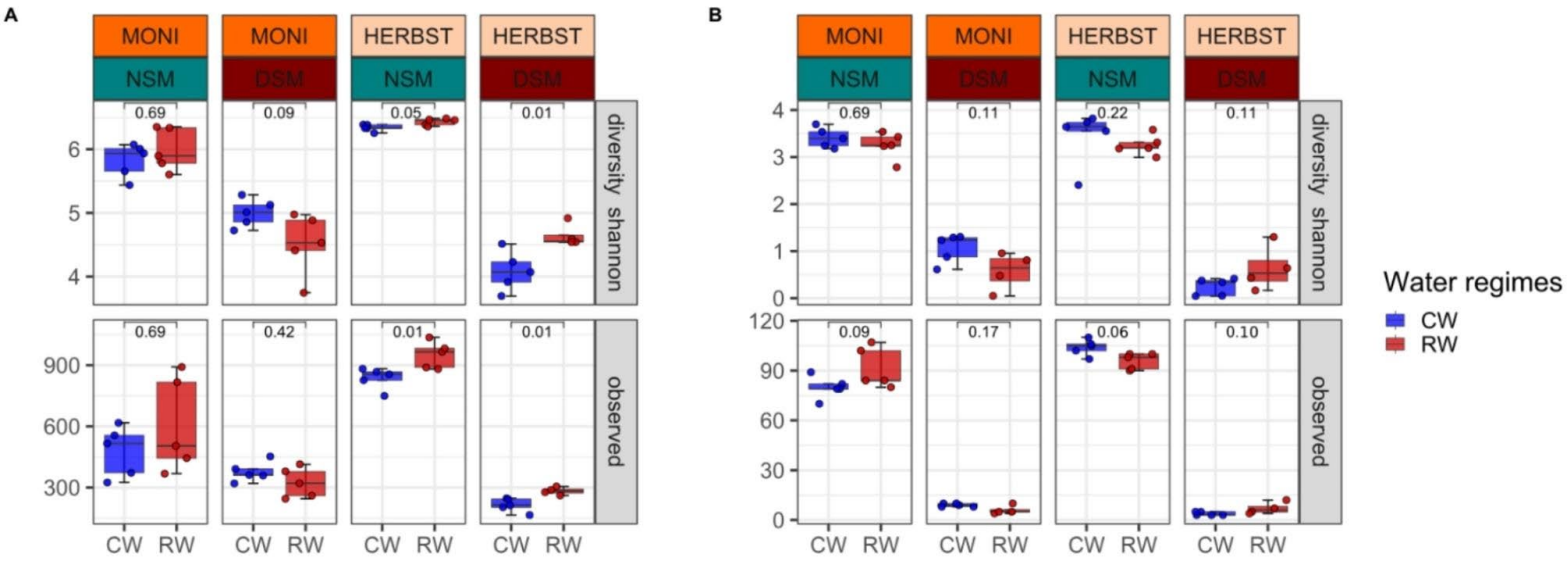
Microbial α-diversity in rhizosphere. Shannon index and observed species of rhizosphere (**A**) bacterial and (**B**) fungal communities of two potato cultivars MONI and HERBST grown either in soil with natural (NSM) or disturbed (DSM) microbiomes were compared between continuous watering (blue) and reduced watering (red). Boxplots display the medians, tops and bottoms of the boxes represent 75th and 25th quartiles, and whiskers outside this range; dots illustrate the individual observations in each sample group. A non-parametric Wilcoxon test (*p* < 0.05, n = 5) was applied to calculate significant differences across sample groups and numbers above the boxes indicate the corresponding *p*-values

Soil autoclaving significantly reduced the starting microbial biomass (Cmic and Nmic) (Supplementary Table S2), microbial diversity (Supplementary Figure S2 and S3), and induced changes in the microbial composition (Supplementary Figure S4). In addition, soil autoclaving increased DOC, DON and inorganic N (NH_4_^+^) (Supplementary Table S2). As expected, pre-treatment of soil resulted in lower bacterial α-diversity of the rhizosphere microbiome in DSM soil, and in rhizosphere samples from both cultivars, MONI (Observed: Wilcoxon test, *p* = 0.00013) and HERBST (Observed: Wilcoxon test, *p* = 2.2e-05) (Table 1 A). In comparison to the continuous watering, reduced watering had no significant effect on bacterial α-diversity of MONI, but consistently resulted in higher diversity in the cultivar HERBST (Fig. 3A).

**Table 1:** Effect of soil pre-treatment (natural (NSM) vs. disturbed (DSM) soil microbiome) on α-diversity (Shannon index, observed species) of A) bacterial and B) fungal communities in the rhizosphere of two potato cultivars (MONI, HERBST). A non-parametric Wilcoxon test (p < 0.05, n = 10) was applied to calculate significant differences across sample groups

A								
Cultivars	α-Diversity	group1	group2	*p*	p.adj	p.format	p.signif	Method
MONI	Shannon	NSM	DSM	0.000011	0.000011	1.10E-05	****	Wilcoxon
MONI	observed	NSM	DSM	0.00684	0.0068	6.80E-03	**	Wilcoxon
HERBST	Shannon	NSM	DSM	0.0000217	0.000022	2.20E-05	****	Wilcoxon
HERBST	observed	NSM	DSM	0.0000217	0.000022	2.20E-05	****	Wilcoxon
**B**								
**Cultivars**	**α-Diversity**	**group1**	**group2**	***p***	**p.adj**	**p.format**	**p.signif**	**Method**
MONI	Shannon	NSM	DSM	0.0000217	0.000022	2.20E-05	****	Wilcoxon
MONI	observed	NSM	DSM	0.0000266	0.00027	2.70E-04	**	Wilcoxon
HERBST	Shannon	NSM	DSM	0.0000217	0.000022	2.20E-05	****	Wilcoxon
HERBST	observed	NSM	DSM	0.000264	0.00026	2.60E-04	****	Wilcoxon

Soil pre-treatment significantly affected fungal α-diversity, being lower in DSM soil for the two cultivars, MONI (Observed: Wilcoxon test, *p* = 0.00027) and HERBST (Observed: Wilcoxon test, *p* = 0.00026) (Table 1B) whereas the effect of the two water regimes was insignificant (Fig. 3B).

PERMANOVA revealed that soil pre-treatment significantly altered bacterial composition in the rhizosphere of both cultivars, MONI (weighted UniFrac, R^2^ = 0.895, *p* = 0.001), and HERBST (weighted UniFrac: R^2^ = 0.866, *p* = 0.001). In contrast, water regimes did not affect the bacterial composition in MONI (weighted UniFrac: R^2^ = 0.011, *p* = 0.17) however, we found a significant interaction of water regimes with soil pre-treatment in HERBST (weighted UniFrac: R^2^ = 0.021, *p* = 0.05). Fungal community was also driven by soil pre-treatment in both cultivars, MONI (weighted UniFrac, F = 9.7419, *p* = 0.001) and HERBST (weighted UniFrac, R^2^ = 0.647, *p* = 0.001) but not influenced by water regimes.

### Microbial responders

**Fig. 4:**
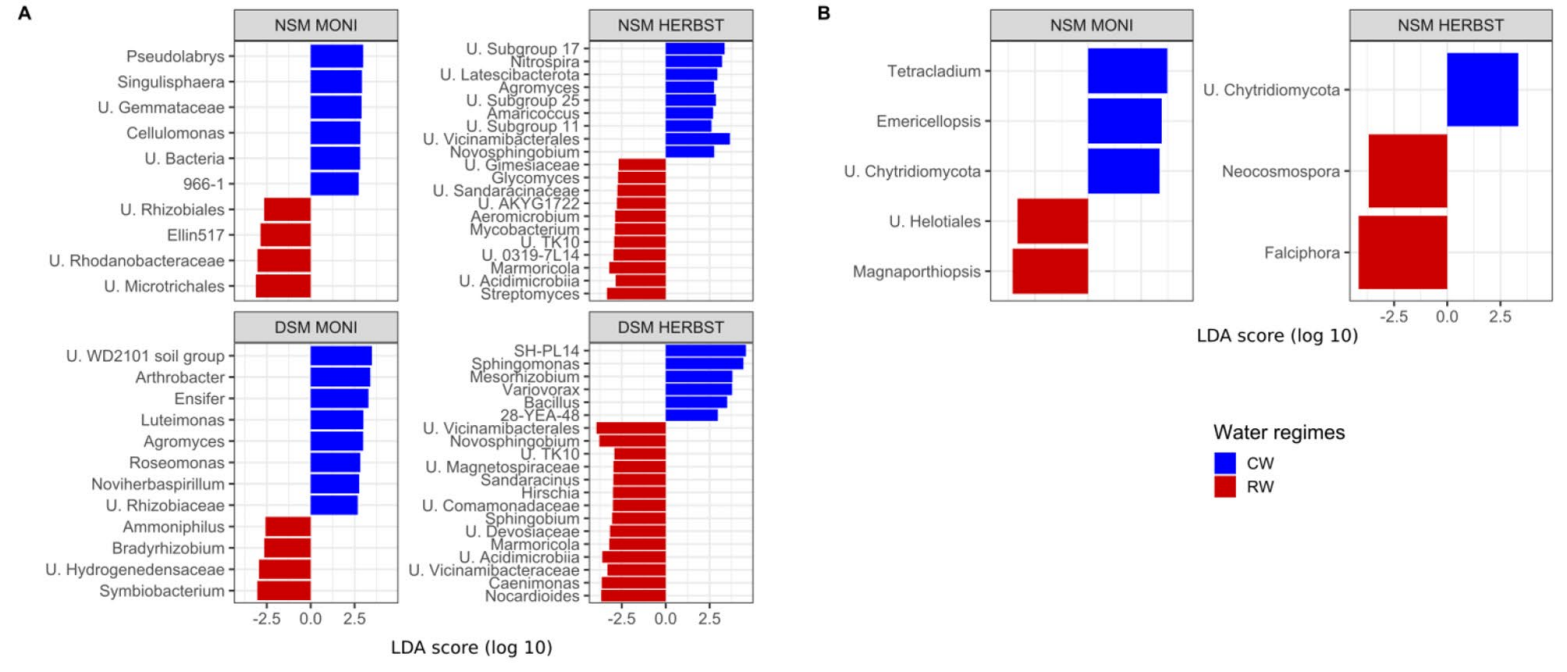
Linear Discriminant Analysis (LDA) combined with Effect Size (LEfSe) plot of (**A**) bacterial and (**B**) fungal genera identified as potential responders of continuous watering (CW, blue) and reduced watering (RW, red) in the rhizosphere of two potato cultivars MONI and HERBST grown in either native soil with natural microbiome (NSM) and autoclaved soil with disturbed microbiome (DSM). Only features meeting an LDA significant threshold ≥ 2.5 and *p*-values < 0.05 for factorial Kruskal–Wallis and pairwise Wilcoxon tests are displayed

We observed that MONI cultivated in NSM soil under continuous watering had a higher abundance of Actinobacteria compared to HERBST in the similar conditions (Supplementary Figure S5). Linear Discriminant Analysis Effect Size (LEfSe) revealed that rhizosphere bacteria and fungi identified as potential responders significantly differed across water regimes, cultivars, and pre-treatment of the soil (Fig. 4). For MONI, Proteobacteria (unclassified Rhizobiales and Rhodanobacteraceae), Verrucomicrobia (Ellin 517) and Actinobacteriota (unclassified Microtrichales) were identified as potential responders in reduced watering samples from NSM soil whereas those were Proteobacteria (*Bradyrhizobium*), Bacillota (*Ammoniphilus*), *Firmicutes* (*Symbiobacterium*) and Hydrogenedentes (unclassified Hydrogenedensaceae) from DSM soil (Fig. 4A). For HERBST grown in NSM soil, responders to the reduced watering included mainly Actinobacteriota lineages (*Streptomyces, Marmoricola, Aeromicrobium, Glycomyces, Mycobacterium*, unclassified Acidimicrobiia and 0319-7L14) and a Myxococcota (unclassified Sandaracinaceae). In DSM soil, although potential responders of reduced watering included fewer Actinobacteriota (*Marmoricola*, *Nocardioides*, Acidimicrobiia unclassified), de novo taxonomic groups such as Proteobacteria (*Novosphingobium, Sphingobium, Hirshia, Caenimonas*, unclassified Devosiaceae and Comamonadaceae), Myxococcota (*Sandaracinus*) and Acidobacteriota (unclassified Vicinamibacterales and Vicinamibacteraceae) were also observed (Fig. 4A). With respect to fungal communities, potential responders of reduced watering in NSM soil consisted of Ascomycota (*Magnaporthiopsis*, and unclassified Helotiales) in MONI and the genera *Falciphora* and *Neocosmospora* in HERBST (Fig. 4B). In DSM soil, no taxa indicative of water regimes were observed in the rhizosphere of these two cultivars.

### Shared microbiomes across soil pre-treatment and water regimes

**Fig. 5:**
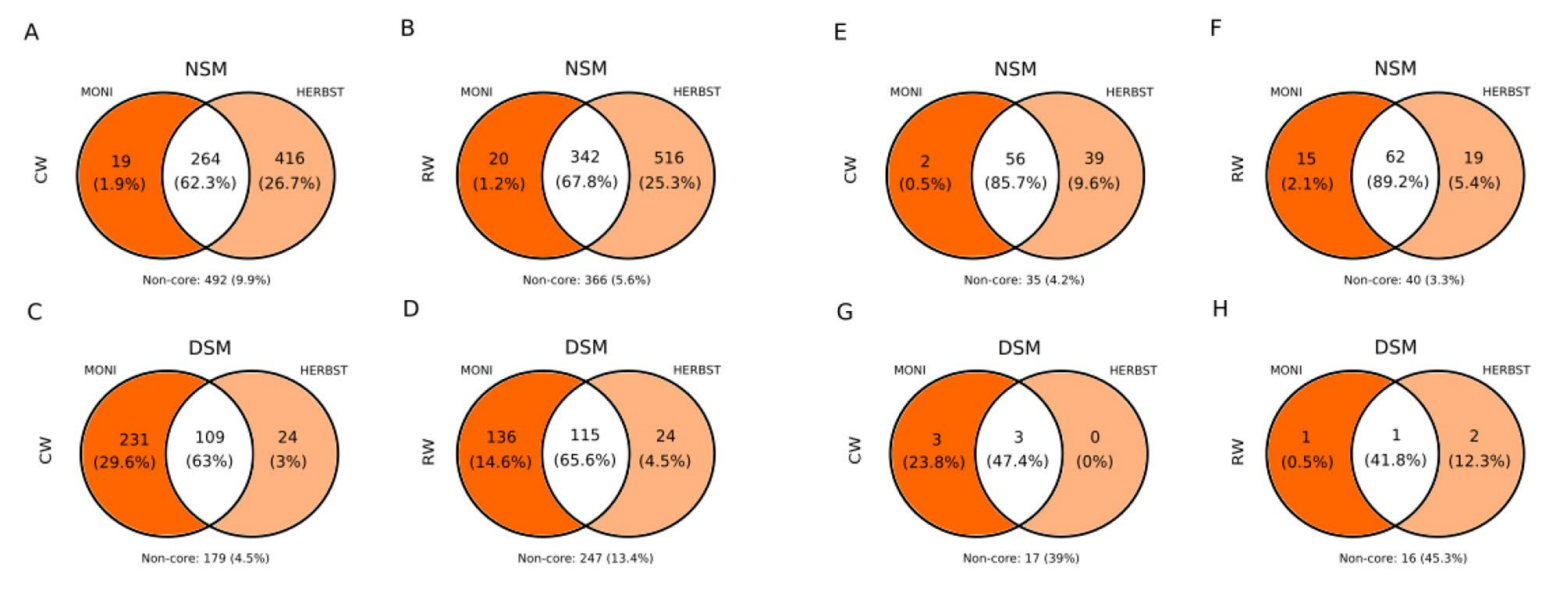
Venn diagram showing the number of unique (colored ellipses) and shared (white ellipses) bacterial and fungal ASVs to two potato cultivars MONI (dark orange) and HERBST (light orange) grown in native soil with natural microbiome (NSM) and autoclaved soil with disturbed microbiome (DSM), under continuous watering (CW; **A**, **C**, **E**, **G**) and reduced watering (RW; **B**, **D**, **F**, **H**). Respective read percentages are indicated in the brackets. Only ASVs found in the 80% of each sample collection with relative abundance of 0.001 were considered for analysis. Non-core represents the part of ASVs and percentage of reads not included in the frequency and abundance cut-offs

**Fig. 6:**
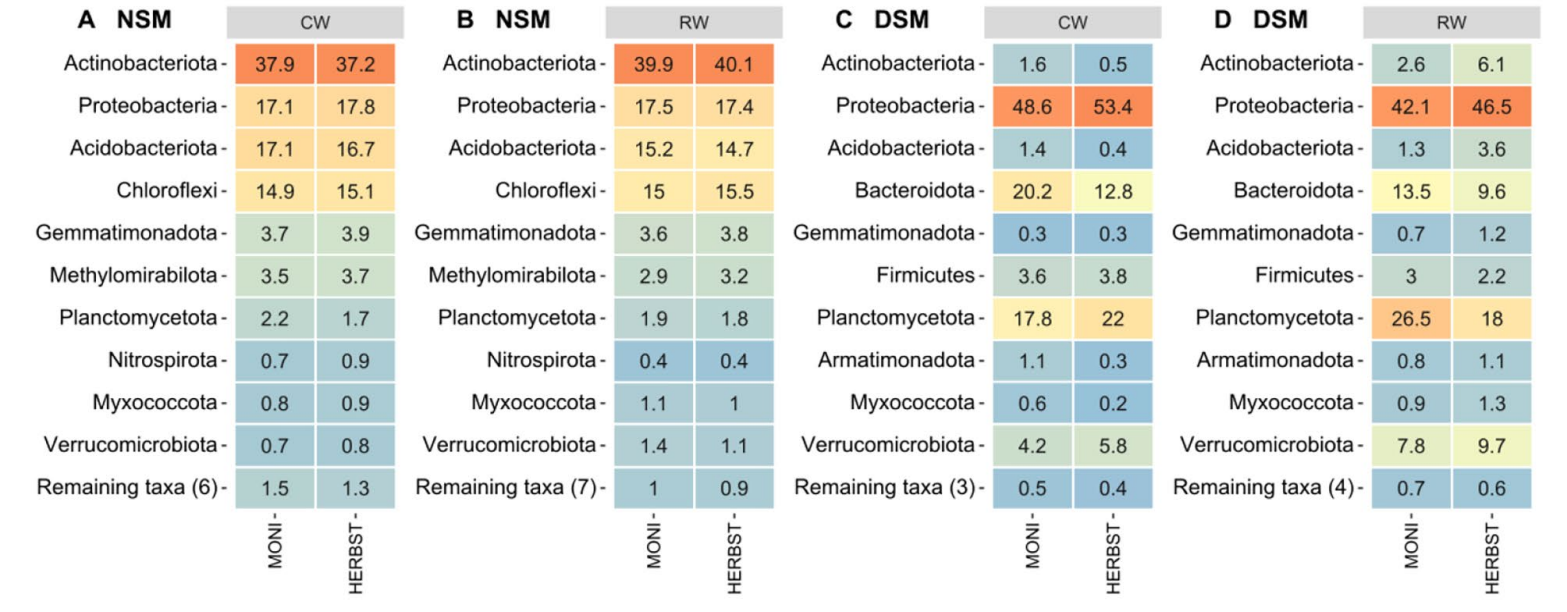
Composition of shared bacterial community. Heatmap displays the top 10 phyla in the shared microbiome of two potato cultivars MONI and HERBST grown in native soil with natural microbiome (NSM) and autoclaved soil with disturbed microbiome (DSM), under continuous watering (CW; **A**, **C**) and reduced watering (RW; **B**, **D**). Numbers in the heatmap indicate the relative abundance of each taxon across sample groups

In NSM soil, both cultivars shared the highest amount of bacterial ASVs under reduced watering, i.e., 342 ASVs representing 67.8% of the total read count (Fig. 5B), compared to 264 ASVs (62.3%) under continuous watering (Fig. 5A). Phylum-based analysis revealed a similar composition of the microbiomes shared by the two cultivars under continuous watering and reduced watering, but slight changes occurred in the relative abundances (Fig. 6A, B). Actinobacteriota were by far the most abundant phylum followed by Proteobacteria, Acidobacteriota and Chloroflexi. Compared to continuous watering, a slight increase and decrease in the proportions of Actinobacteriota and Acidobacteriota, respectively, was observed under reduced watering, whereas Proteobacteria and Chloroflexi remained relatively stable (Fig. 6A, B). At the genus level, *Gaiella*, *Arthrobacter, Nocardioides* and unclassified Gaiellales, MB-A2-108, 67 − 14, were the most represented Actinobacteriota; *Hyphomicrobium* and *Ellin6067* in Proteobacteria; unclassified Vicinamibacterales and Vicinamibacteraceae in Acidobacteriota and unclassified KD4-96, Gitt-GS-136, JG30-KF-CM45 in Chloroflexi (Supplementary Figure S6A, B). In DSM soil, MONI and HERBST also shared a microbiome under continuous watering (109 ASVs accounting for 63% total read count) and reduced watering (115 ASVs representing 65.3% total read count) (Fig. 5C, D). Soil pre-treatment overall changed the composition of the shared microbiome at the phylum level in DSM soil by increasing the relative abundances of Proteobacteria, Planctomycetes, Bacteroidota, Firmicutes and Verrucomicrobia at the expense of Actinobacteriota, Acidobacteriota, Chloroflexi and Gemmatimonadota (Fig. 6C, D). For HERBST cultivated in DSM soil, Actinobacteriota increased under reduced watering, but their proportion remained marginal compared to the corresponding treatment in NSM soil. At the genus level, *SH-PL14* was differentially abundant, especially enriched in MONI and depleted in HERBST under reduced watering treatment. *Sphingomonas* also decreased in HERBST under reduced watering but not in MONI (Supplementary Figure S6C, D). Comparing the two cultivars, the number of core ASVs was relatively consistent in MONI rhizosphere regardless of the soil pre-treatment (Table 2), while in HERBST, core ASVs were considerably lower in DSM soil compared to NSM soil (Table 2).

**Table 2:** Summary of bacterial core ASVs in the rhizosphere of two potato cultivars MONI and HERBST across soil pre-treatment and water regimes

Cultivars	MONI				HERBST			
Soil pre-treatment	NSM		DSM		NSM		DSM	
Water regimes	CW	RW	CW	RW	CW	RW	CW	RW
**Core ASVs**	283	362	340	251	680	858	133	139

**Fig. 7:**
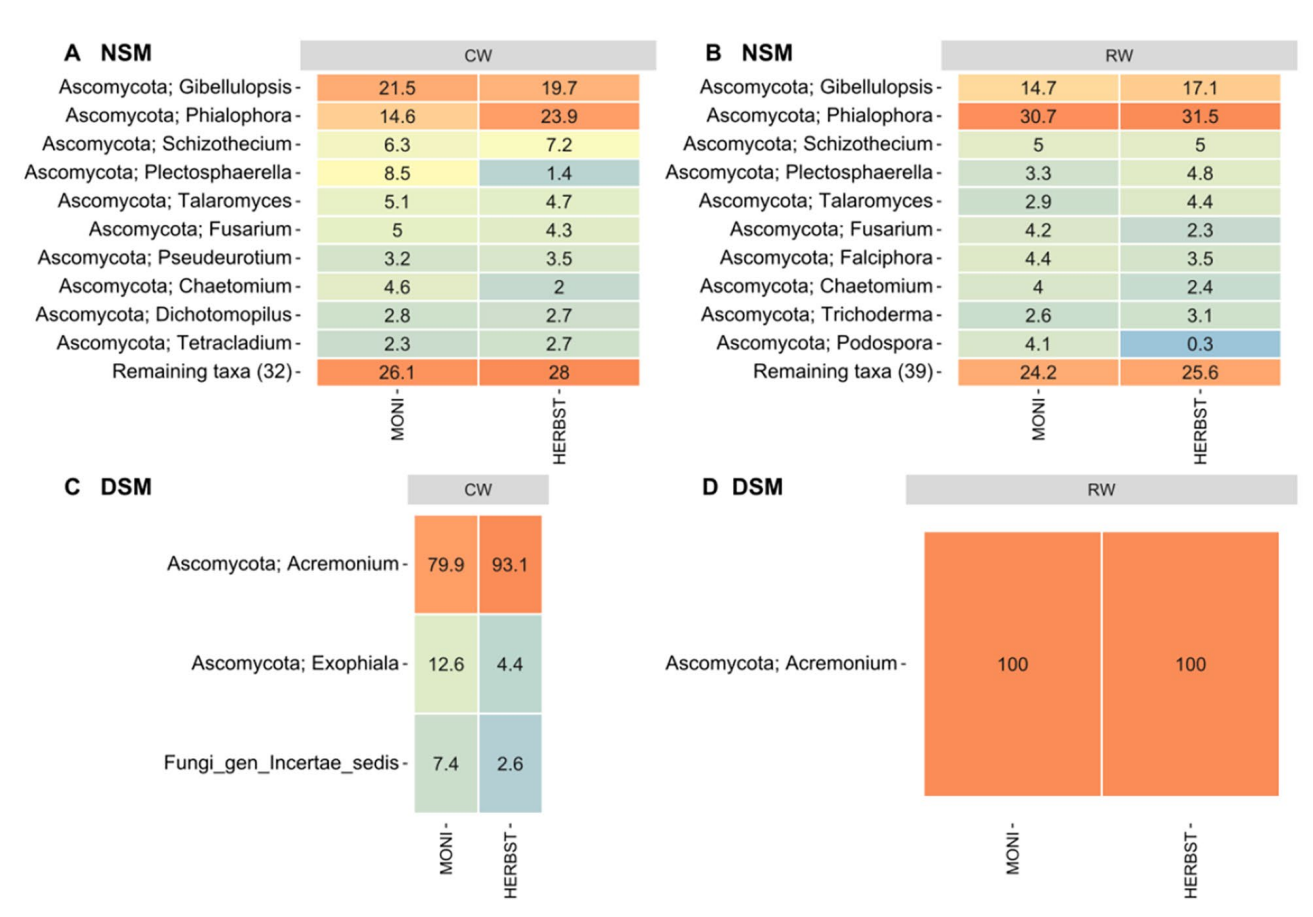
Composition of shared fungal community. Heatmap displays the top 10 genera in the shared microbiome of two potato cultivars MONI and HERBST grown in native soil with natural microbiome (NSM) and autoclaved soil with disturbed microbiome (DSM), under continuous watering (CW; A, C) and reduced watering (RW; B, D). Numbers in the heatmap indicate the relative abundance of each taxon across sample collections

In NSM soil, the two cultivars exhibited overlapping fungal ASVs, with a slightly higher number observed under reduced watering (62 ASVs accounting for 89.2% of the total read count) compared to continuous watering (56 ASVs representing 85.7% of the total read count) (Fig. 5E, F). Shared ASVs were mostly assigned to the phylum Ascomycota (Supplementary Figure S7). At the genus level, *Podospora*, *Trichoderma* and *Falciphora* were among the top 10 taxa under reduced watering, but not under continuous watering (Fig. 7A, B). Interestingly, *Podospora* and *Falciphora* were not found under continuous watering (Supplementary Figure S7E). *Phialophora* increased in proportion under reduced watering compared to continuous watering in both cultivars, whereas, *Gibellulopsis* remained stable in HERBST, but decreased in MONI under reduced watering relative to continuous watering (Fig. 7A, B). In DSM soil, shared fungal ASVs were strongly reduced irrespective of water regimes (Fig. 5G-H). Under continuous watering, 3 ASVs were shared, belonging to the genera *Acremonium* and *Exophiala*; under reduced watering, it was only 1 ASV, namely *Acremonium* (Fig. 7C, D).

## Discussion

We investigated the rhizosphere microbiome of potato under reduced soil moisture. To understand the influence of the genetic variability, we used two cultivars of potato with contrasting levels of drought tolerance. Lastly, the contribution of soil conditions in our system was assessed through the manipulation of the soil microbial biomass, diversity and composition by soil autoclaving.

Interestingly, soil pre-treatment had no significant influence on growth parameters of both cultivars under continuous watering. However, the combination of DSM treatment with reduced watering significantly reduced the root length and root fresh weight in the sensitive cultivar HERBST but did not affect root length of the resistant cultivar MONI. Roots play a critical role in plant adaptation to abiotic stress including water acquisition, nutrient uptake to the plant and interaction with microorganisms in the rhizosphere [65, 66]. Furthermore, increased productivity of plants under drought has been correlated with key morphological traits influencing the root length and surface area of root systems [67]. An inverse relationship (i.e., the larger the root system, the smaller the yield decline) has been observed under drought, suggesting that cultivars with deep root systems may be drought-resistant [68]. In a previous study [69], it was shown that two drought tolerant cultivars of potato (GWIAZDA and TAJFUN), developed elongated roots when challenged with drought, whereas in drought susceptible cultivars (OBERON and CEKIN), the root length was unchanged under drought. These observations are not consistent with our results, potentially due to the choice of cultivars, the magnitude of the drought stress, pot size and the plant growth stage at the time of the treatment (tuber initiation). Potato responses towards drought were reported to vary with phenological stage, genotype, and stress severity [13, 70]. Nevertheless, another study showed a reduction in root length under drought in potato [71], supporting our findings from the cultivar HERBST. However, this reduction was only visible in our study when plants were cultivated in the soil with strongly reduced microbial diversity, suggesting a potential positive relationship between soil microbiome diversity and root growth.

### Response of the rhizosphere microbiome to water reduction is cultivar dependent

Many studies have previously demonstrated that soil acting as the microbial reservoir is the main contributor to the rhizosphere microbiome [29–31]. This has been confirmed in our study by the strong impact soil pre-treatment had on the rhizosphere microbial communities (bacteria and fungi) of potato. Differences observed between NSM and DSM rhizosphere microbiomes can be explained by their respective source soils. In fact, soil autoclaving significantly reduced the starting microbial biomass (Cmic and Nmic), microbial diversity, and induced changes in the microbial composition. In addition, soil autoclaving increased inorganic N (NH_4_^+^), DON and DOC. As previously observed elsewhere, increase in DOC following autoclaving reflect the release of dissolved nutrients from dead cells into the soil [72]. Therefore, DSM soil was enriched in carbon sources which can be easily used for growth by hetero- and copiotrophic microbes, and which induce a complete restructuring of microbial communities. As discussed below, this may notably favour the rapid colonization of bacteria such as Proteobacteria or Bacteroidota in the plant rhizosphere [73]. Nevertheless, it is important to emphasize that this study did not intend to investigate the effect of soil autoclaving on the subsequently acquired rhizosphere microbiome. We rather employed this technique to manipulate the diversity of the natural soil microbiome in order to study the interaction between two potato cultivars with different drought tolerance and differently diverse rhizosphere microbiota, specifically under reduced soil moisture.

### Soil with natural microbiome (NSM)

Increasing evidence supports the role of root associated microbial communities in plant responses to environmental stresses. We hypothesized that when drought tolerance is mediated in potato by the recruitment of beneficial microbes, the rhizosphere microbiome would be affected in a cultivar-specific manner under reduced soil moisture, with the resistant cultivar MONI exhibiting a more adaptive microbiome under drought than the susceptible cultivar HERBST (**H1**). In NSM soil, MONI showed a stable rhizosphere microbiome regardless of the water regimes and potentially recruited less but drought tolerant microbes such as Rhizobiales [38] under reduced watering. We witnessed a significant response to the reduced watering in HERBST rhizosphere microbiome (diversity and composition), resulting in potential recruitment of well-known drought-responsive taxa mainly in the Actinobacteriota phylum [38, 40, 41], and the root endophytic fungus *Falciphora* [74]. Thus, although there is a host-dependent differentiation in the rhizosphere microbiome under reduced watering, we found no support for **H1**. Previously, tomato cultivars thoroughly selected for drought tolerance were used to compare their rhizosphere microbiome under full and deficit irrigation regimes [39]. As observed for MONI, the tolerant tomato cultivar showed unchanged bacterial and fungal diversity irrespective of the irrigation treatment, which the authors attributed to a buffering effect exerted by the tolerant cultivar on its rhizosphere [39]. In contrast, a significant increase in bacterial diversity during deficit irrigation was shown for the sensitive tomato cultivar [39], consistent with our observations in HERBST rhizosphere. Specialized metabolites produced by *Solanum* plants, especially α-tomatine in tomato and α-solanine in potato [75], two glycoalkaloids with known antimicrobial effects [76, 77] have been suggested to reduce the growth of many bacterial families in soil [78]. Increased microbial α-diversity suggests that reduced watering could interfere with the production of these metabolites in drought-sensitive cultivars of Solanaceae.

Cultivar-dependent response to reduced watering observed in our study can likely be attributed to root exudates, which are recognized as the primary factor in shaping the composition of the rhizosphere microbiome [79–82]. Root exudates do not only depend on plant genotypes [83, 84], their quantity and quality may also change under drought conditions [34, 85, 86]. For example, increase in organic acids in the root exudates under moderate drought conditions stimulated microbial activities in the rhizosphere [87, 88]. Furthermore, Actinobacteria have been positively correlated with some of the exuded organic acids such as salicylic acid and Gamma-aminobutyric acid (GABA) [89]. Nevertheless, the underlying mechanisms responsible for the active selection of monoderm bacteria, such as Actinobacteriota, under drought as demonstrated elsewhere [36–38, 40, 41, 90] and confirmed in HERBST rhizosphere, warrant further investigation.

Interestingly, in NSM soil, in the rhizosphere of the resistant cultivar MONI grown under continuous watering Actinobacteria were high in relative abundance, whereas in the sensitive cultivar HERBST, they were only enriched under reduced watering. These findings are in line with a study using sugarcane, where the authors demonstrated that when cultivated under optimal watering, the rhizosphere of a tolerant cultivar was rich in Actinobacteria and Alphaproteobacteria, while the sensitive cultivar was enriched in Actinobacteria solely under drought [36]. However, for other plants species, different response patterns were found including alfalfa where in the rhizosphere of the tolerant cultivar grown under well-watered conditions, Proteobacteria and Bacteroidetes were prevalent and increased under drought in the sensitive cultivar [35]. These observations support the assumption, that the observed abundance of drought-tolerant bacteria in the rhizosphere of drought-tolerant plant cultivars under optimal water conditions as well as under drought cannot be generalized but are plant specific and recruiting mechanisms may differ accordingly [36]. Under drought, Rhizobiales were enriched in MONI rhizosphere whereas in other studies, it was Acidobacteria in tolerant alfalfa [35], and Bacilli in tolerant sugarcane [36]. This may suggest that different plant families have different strategies and there is no uniform pattern of recruitment in drought tolerant cultivars. In contrast to the resistant cultivar, we observed a stronger response to reduced watering in the rhizosphere of the sensitive cultivar HERBST. Notably, this led to the selection of several Actinobacteria members. Our results align with previous observations in susceptible sugarcane and broomcorn millet cultivars [36, 37], and suggest that the rhizosphere of sensitive cultivars, being less prepared under optimal watering, are much more affected by drought disturbances, which consequently drive the significant changes in the rhizosphere microbiome. Actinobacteria recruitment and their activity are stimulated by changes in root metabolism induced by drought stress [41]. Moreover, this selection of Actinobacteria was more pronounced as plant-microbe interactions increased (endosphere > rhizosphere > bulk soil) [40], implying that they play an important role in plant response towards drought. Drought responsive Actinobacteria in HERBST rhizosphere included *Streptomyces*, *Glycomyces*, *Marmoricola*, *Aeromicrobium*, *Mycobacterium* and others. While *Streptomyces* was enriched in drought-treated potato rhizosphere [90], an inoculation assay of a strain of this genus to *Sorghum bicolor* reported its effective colonization of plant and root development under drought [41]. One of drought tolerance mechanisms by which plants mitigate damages of reactive oxygen species (ROS) is the release of hydrogen peroxide (H_2_O_2_) [91], which has been associated with the preservation of apical root growth during drought [92]. The ability of many *Streptomyces* isolates to mitigate ROS damage in plants [93, 94] may potentially be linked to their recruitment under drought. Although some *Streptomyces* species were reported as plant pathogens [95, 96], our results indicate that the recruitment of this genus in HERBST rhizosphere may potentially have positive implications for plant fitness especially root growth under reduced soil moisture. Another mode of action of microbial-mediated response is the modulation of phytohormone levels [97, 98] such as ethylene. The latter plays a critical role in plant responses to stress, especially at the root level [99, 100]. Many drought-responsive Actinobacteria contain the *acdS* gene encoding the enzyme 1-amino cyclopropane-1-carboxlate deaminase (ACCd) [44]. This enzyme can effectively degrade the direct ethylene precursor (ACC) to ammonia and α-ketoglurate, thereby reducing ethylene biosynthesis under drought. The analysis of *acdS* gene showed an enrichment of *Marmoricola* in the rhizosphere of drought stressed barley [44]. The selection of this genus in HERBST rhizosphere suggests that the reduction of ethylene biosynthesis may be associated with root growth in HERBST, as this mechanism has been demonstrated under drought, flooding, heat, cold, pathogen colonization etc. [101]. Interestingly, *Falciphora* was observed as responders to reduced watering in HERBST rhizosphere. This genus was previously reported as plant endophyte and its co-cultivation with *Arabidopsis thaliana* improved lateral root growth via regulation of auxin biosynthesis, signalling and transport in plant [74]. The selection of *Falciphora* along HERBST rhizosphere under reduced watering may underline the putative role of root endophytes in plant growth particularly under abiotic stress [102]. Potato is often regarded as a drought-sensitive crop, mainly attributed to its shallow and sparse root system [12]. Taken together, our results suggest that both cultivars grown in NSM soil recruited beneficial microbes that potentially support root growth under reduced soil moisture. Our findings corroborate reports indicating that fungal communities generally exhibit a high level of drought tolerance, as only a limited number of fungal taxa exhibited responses to the reduced watering [49]. Nevertheless, it is evident that the taxa influenced by the reduced soil moisture were either directly affected by drought (saprotrophs) or indirectly by their root associated lifestyle [103], suggesting a differential drought tolerance also in fungal communities as reported by [104].

### Soil with disturbed microbiome (DSM)

We hypothesized that when the rhizosphere microbiome plays a dominant role in drought resistance of the two potato cultivars, then a reduction of the soil microbial diversity would result in lower drought tolerance under reduced watering in both cultivars (**H2**). Surprisingly, despite the reduction of microbial diversity in the source soil, the rhizosphere microbiome (bacteria and fungi) of the resistant cultivar MONI was unaffected (α and β diversity) by the reduced watering regime. Furthermore, a restricted number of bacterial responders to reduced watering was found in the rhizosphere of MONI in DSM soil, similarly to NSM soil. This stability in the rhizosphere could have supported growth parameters of MONI and perhaps increased its leaf dry weight. However, the sensitive cultivar HERBST showed significant changes in bacterial communities under reduced watering when plants were grown in DSM soil. Moreover, the latter induced the recruitment of other potential microbial responders such as Proteobacteria, Acidobacteriota and fewer Actinobacteriota under reduced watering. Acidobacteriota generally decrease in relative abundance under drought [38, 40], suggesting that some of members in this phylum are sensitive to drought. We argue that this substitution, however incomplete, of drought tolerant Actinobacteriota by Proteobacteria and Acidobacteriota could be one of the reasons behind the significant reduction in root length of HERBST cultivated under reduced watering. Therefore, since the plants were differentially affected by reduced watering in DSM soil, we reject **H2**. Interestingly, we were able to find that in the two potato cultivars, none of plant growth parameters was affected under continuous watering, despite the strong effect of soil pre-treatment on rhizosphere microbial communities. These findings suggest that plants may have a more pronounced interaction with their rhizosphere microbiome when experiencing reduced soil moisture than under optimal watering.

In contrast to the bacterial communities, fungi were not affected by water regimes and no potential responsive taxa were detected in the rhizosphere for either cultivar in DSM soil. In line with our observations, previous studies reported a less pronounced and even non-existent effect of drought on the structure of fungal communities in soil and root associated microbiomes [40, 46–48]. This may partly be attributed to fungal spores which are highly persistent towards drought stress. In this respect also Streptomycetes and other Actinobacteria must be discussed as it cannot be excluded that their high relative abundance in the drought affected soils is partly related to their potential to form inactive forms, which cannot be differentiated from vegetative cells using an DNA based metabarcoding approach. Fungal communities in our study were less diverse than bacterial communities in NSM soil, as previously showed elsewhere [39, 49], and the soil autoclaving had almost eradicated them from the plant rhizosphere. This could lead to the non-selection of drought responsive fungal taxa such as *Falciphora* in the rhizosphere of HERBST, which we suggest may increase the susceptibility of this cultivar in conditions of reduced watering.

### Cultivars had a shared microbiome across soil pre-treatment and water regimes

Our study reveals the existence of shared microbiomes between the different sample groups providing evidence in the two potato cultivars of similar patterns in their rhizosphere microbiome composition, irrespective of the treatments. Regarding the bacterial communities, it appears that Actinobacteriota play a crucial role as the main drivers of the response to the water regimes in the native soil. In addition to the reasons mentioned earlier in this discussion, these bacteria possess physiological (degrading recalcitrant compounds, sporulation) and structural characteristics (thicker peptidoglycan cell wall) [34] that allow them to thrive in dry environments. Thus, their strong interaction with the host rhizosphere may participate in the alleviation of stress in the two potato cultivars. *Gaiella*, *Nocardioides*, *Arthrobacter*, *Marmoricola* and *Streptomyces* were the most shared Actinobacteriota. *Gaiella* was found very abundantly in soil with reduced moisture and is involved in nitrogen cycling [105]. The other genera *Nocardioides* [106], *Arthrobacter* [107], *Marmoricola* [44] and *Streptomyces* [41, 90] have been reported as plant growth promoting rhizobacteria with ability to produce auxins, siderophore and many other compounds [108]. Members of the fungal genera *Falciphora* (often associated with an endophytic lifestyle [102]) and *Trichoderma* (with strain specific drought tolerance [109]) increased in relative abundance and have been previously recognized to encompass drought tolerant strains that potentially could sustain plant growth under stressful conditions. However, this assumption needs further investigations.

We also found that the rhizosphere of MONI had a relatively consistent number of bacterial core ASVs compared to HERBST, irrespective of soil pre-treatment and water regimes. Another striking finding was that DSM soil pre-treatment led to a complete restructuring of the shared microbial communities of the rhizosphere. This restructuring was characterized by Proteobacteria and other fast-growing bacterial taxa emerging as the main drivers of the response to water regimes. Specifically, we observed a substantial decrease in the relative abundance of Actinobacteriota, Chloroflexi, Acidobacteriota among many others, while there was a marked increase in Proteobacteria, Bacteroidota, Verrucomicrobiota, and Firmicutes. We suggest that the resistant cultivar MONI, which demonstrated the ability to recruit members of Proteobacteria and Verrucomicrobiota (LEfSe analysis) in NSM soil, could be able to adapt quickly in DSM soil even when subjected to reduced watering. In contrast, the sensitive cultivar HERBST, which showed a preference for Actinobacteriota, was not able to establish in DSM soil, an interaction as strong as that observed in the NSM soil under reduced watering. Furthermore, drought-responsive fungal taxa found in NSM soil were not observed in the shared microbiomes in DSM soil. Collectively, reduction in Actinobacteriota combined with absence of drought responsive fungi in the shared microbiomes may explain reduced root length of HERBST under reduced soil moisture.

## Conclusion

In summary, we demonstrate that potato cultivars of different drought tolerance engage with distinct microbial players when exposed to the same stress conditions, suggesting a link between host-microbial differentiation and the plant capacity to respond to drought. We found that the resistant cultivar had already drought tolerant taxa in its rhizosphere under continuous watering and therefore moderately changed its microbiome under reduced watering. Conversely, the sensitive cultivar selected these drought tolerant taxa only when reduced watering was imposed. Our study contributes to reducing knowledge gaps regarding the interactions of potato plants and their rhizosphere microbiomes under reduced soil moisture. However, a number of spore-forming taxa was found in the microbiome. This points out the need of using metagenomic and metatranscriptomic approaches in the future to assess the activity of microbial communities in the rhizosphere of potato plants under drought. Further research work on these cultivars (MONI and HERBST) considering factors such as plant developmental stage, soil type, and farming system, all of which can influence microbial community composition, will provide valuable insights into the role of the rhizosphere microbiome in sustaining potato growth and production under drought stress in the field condition with complex environmental interactions. In general, our data clearly demonstrate the importance of both factors soil microbiome and plant genotype for the development of a stable microbial community in the rhizosphere which mitigate abiotic and most likely also biotic stressors. Therefore, elucidating the underlying mechanisms how plants recruit their microbiome from soil might be an important issue for future research, as this information might be of high value for future targeted plant breeding programs ensuring the development of a “healthy” plant microbiome belowground.

### Electronic supplementary material

Below is the link to the electronic supplementary material.

Supplementary Material 1

## Data Availability

The datasets supporting the conclusions of this article are available in the NCBI repository, in the BioProject PRJNA978426.

## List of Abbreviations

NSM: Natural soil microbiome
DSM: Disturbed soil microbiome
HERBST: Cultivar HERBSTFREUDE
WHC: Water holding capacity
ASV: Amplicon sequence variant

## References

[1] Fahad S, Bajwa AA, Nazir U, Anjum SA, Farooq A, Zohaib A, Sadia S, Nasim W, Adkins S, Saud S, Ihsan MZ, Alharby H, Wu C, Wang D, Huang J (2017). Crop production under drought and heat stress: plant responses and management options. Front Plant Sci.

[2] Yaseen R, Aziz O, Saleem MH, Riaz M, Zafar-ul-Hye M, Rehman M, Ali S, Rizwan M, Alyemeni MN, El-Serehy HA, Al-Misned FA, Ahmad P (2020). Ameliorating the drought stress for wheat growth through application of ACC-deaminase containing rhizobacteria along with biogas slurry. Sustain.

[3] Alam H, Khattak JZK, Ksiksi TS, Saleem MH, Fahad S, Sohail H, Ali Q, Zamin M, El-Esawi MA, Saud S, Jiang X, Alwahibi MS, Alkahtani J (2021). Negative impact of long-term exposure of salinity and drought stress on native Tetraena mandavillei L. Physiol Plant.

[4] Yasmin H, Bano A, Wilson NL, Nosheen A, Naz R, Hassan MN, Ilyas N, Saleem MH, Noureldeen A, Ahmad P, Kennedy I (2022). Drought-tolerant Pseudomonas sp. showed differential expression of stress-responsive genes and induced drought tolerance in *Arabidopsis thaliana*. Physiol Plant.

[5] AL-Huqail AA, Saleem MH, Ali B, Azeem M, Mumtaz S, Yasin G, Marc RA, Ali S (2023). Efficacy of priming wheat (*Triticum aestivum*) seeds with a benzothiazine derivative to improve drought stress tolerance. Funct Plant Biol.

[6] Zhang H, Sun X, Dai M (2022). Improving crop drought resistance with plant growth regulators and rhizobacteria: mechanisms, applications, and perspectives. Plant Commun.

[7] FAOSTAT. Crops and livestock products-trade. 2021. https://www.fao.org/faostat/en/#data/QCL. Accessed 15 May 2023.

[8] Sprenger H, Rudack K, Schudoma C, Neumann A, Seddig S, Peters R, Zuther E, Kopka J, Hincha DK, Walther D, Köhl K (2015). Assessment of drought tolerance and its potential yield penalty in potato. Funct Plant Biol.

[9] Sprenger H, Kurowsky C, Horn R, Erban A, Seddig S, Rudack K, Fischer A, Walther D, Zuther E, Köhl K, Hincha DK, Kopka J (2016). The drought response of potato reference cultivars with contrasting tolerance. Plant Cell Environ.

[10] Stark JC, Love SL, King BA, Marshall JM, Bohl WH, Salaiz T (2013). Potato cultivar response to seasonal drought patterns. Am J Potato Res.

[11] Nasir MW, Toth Z (2022). Effect of drought stress on potato production: a review. Agronomy.

[12] Yuan BZ, Nishiyama S, Kang Y (2003). Effects of different irrigation regimes on the growth and yield of drip-irrigated potato. Agric Water Manag.

[13] Monneveux P, Ramírez DA, Pino MT (2013). Drought tolerance in potato (*S. Tuberosum* L.). Can we learn from drought tolerance research in cereals?. Plant Sci.

[14] Obidiegwu JE, Bryan GJ, Jones HG, Prashar A (2015). Coping with drought: stress and adaptive responses in potato and perspectives for improvement. Front Plant Sci.

[15] Joshi M, Fogelman E, Belausov E, Ginzberg I (2016). Potato root system development and factors that determine its architecture. J Plant Physiol.

[16] Stalham MA, Allen EJ, Rosenfeld AB, Herry FX (2007). Effects of soil compaction in potato (*Solanum tuberosum*) crops. J Agric Sci.

[17] Spitters CJT, Schapendonk AHCM (1990). Evaluation of breeding strategies for drought tolerance in potato by means of crop growth simulation. Genet Asp Plant Miner Nutr.

[18] Tiwari JK, Buckseth T, Zinta R, Bhatia N, Dalamu D, Naik S, Poonia AK, Kardile HB, Challam C, Singh RK, Luthra SK, Kumar V, Kumar M (2022). Germplasm, breeding, and genomics in potato improvement of biotic and abiotic stresses tolerance. Front Plant Sci.

[19] Berg G (2009). Plant-microbe interactions promoting plant growth and health: perspectives for controlled use of microorganisms in agriculture. Appl Microbiol Biotechnol.

[20] Ngumbi E, Kloepper J (2016). Bacterial-mediated drought tolerance: current and future prospects. Appl Soil Ecol.

[21] Delgado-Baquerizo M, Maestre FT, Reich PB, Jeffries TC, Gaitan JJ, Encinar D, Berdugo M, Campbell CD, Singh BK (2016). Microbial diversity drives multifunctionality in terrestrial ecosystems. Nat Commun.

[22] Philippot L, Raaijmakers JM, Lemanceau P, Van Der Putten WH (2013). Going back to the roots: the microbial ecology of the rhizosphere. Nat Rev Microbiol.

[23] Pascale A, Proietti S, Pantelides IS, Stringlis IA (2020). Modulation of the root microbiome by plant molecules: the basis for targeted dsease suppression and plant growth promotion. Front Plant Sci.

[24] Berendsen RL, Pieterse CMJ, Bakker PAHM (2012). The rhizosphere microbiome and plant health. Trends Plant Sci.

[25] Li J, Wang C, Liang W, Liu S (2021). Rhizosphere microbiome: the emerging barrier in plant-pathogen interactions. Front Microbiol.

[26] Yang C, Yue H, Ma Z, Feng Z, Feng H, Zhao L, Zhang Y, Deakin G, Xu X, Zhu H, Wei F (2022). Influence of plant genotype and soil on the cotton rhizosphere microbiome. Front Microbiol.

[27] Garcia J, Kao-Kniffin J (2018). Microbial group dynamics in plant rhizospheres and their implications on nutrient cycling. Front Microbiol.

[28] Vandenkoornhuyse P, Quaiser A, Duhamel M, Le Van A, Dufresne A (2015). The importance of the microbiome of the plant holobiont. New Phytol.

[29] Veach AM, Morris R, Yip DZ, Yang ZK, Engle NL, Cregger MA, Tschaplinski TJ, Schadt CW (2019). Rhizosphere microbiomes diverge among *Populus trichocarpa* plant-host genotypes and chemotypes, but it depends on soil origin. Microbiome.

[30] Liu F, Hewezi T, Lebeis SL, Pantalone V, Grewal PS, Staton ME (2019). Soil indigenous microbiome and plant genotypes cooperatively modify soybean rhizosphere microbiome assembly. BMC Microbiol.

[31] Inceoǧlu Ö, Salles JF, van Elsas JD (2012). Soil and cultivar type shape the bacterial community in the potato rhizosphere. Microb Ecol.

[32] Gargallo-Garriga A, Preece C, Sardans J, Oravec M, Urban O, Peñuelas J (2018). Root exudate metabolomes change under drought and show limited capacity for recovery. Sci Rep.

[33] Chen Y, Yao Z, Sun Y, Wang E, Tian C, Sun Y, Liu J, Sun C (2022). Current studies of the effects of drought stress on root exudates and rhizosphere microbiomes of crop plant species. Int J Mol Sci.

[34] Naylor D, Coleman-Derr D (2018). Drought stress and root-associated bacterial communities. Front Plant Sci.

[35] Fan W, Tang F, Wang J, Dong J, Xing J, Shi F (2023). Drought-induced recruitment of specific root-associated bacteria enhances adaptation of alfalfa to drought stress. Front Microbiol.

[36] Liu Q, Xie S, Zhao X, Liu Y, Xing Y, Dao J, Wei B, Peng Y, Duan W, Wang Z (2021). Drought sensitivity of sugarcane cultivars shapes rhizosphere bacterial community patterns in response to water stress. Front Microbiol.

[37] Na X, Cao X, Ma C, Ma S, Xu P, Liu S, Wang J, Wang H, Chen L, Qiao Z (2019). Plant stage, not drought stress, determines the effect of cultivars on bacterial community diversity in the rhizosphere of broomcorn millet (*Panicum miliaceum* L). Front Microbiol.

[38] Santos-Medellín C, Edwards J, Liechty Z, Nguyen B, Sundaresan V (2017). Drought stress results in a compartment-specific restructuring of the rice root-associated microbiomes. MBio.

[39] Gaete A, Pulgar R, Hodar C, Maldonado J, Pavez L, Zamorano D, Pastenes C, González M, Franck N, Mandakovic D (2021). Tomato cultivars with variable tolerances to water deficit differentially modulate the composition and interaction patterns of their rhizosphere microbial communities. Front Plant Sci.

[40] Naylor D, Degraaf S, Purdom E, Coleman-Derr D (2017). Drought and host selection influence bacterial community dynamics in the grass root microbiome. ISME J.

[41] Xu L, Naylor D, Dong Z, Simmons T, Pierroz G, Hixson KK, Kim YM, Zink EM, Engbrecht KM, Wang Y, Gao C, DeGraaf S, Madera MA, Sievert JA, Hollingsworth J, Birdseye D, Scheller HV, Hutmacher R, Dahlberg J, Jansson C, Taylor JW, Lemaux PG, Coleman-Derr D (2018). Drought delays development of the sorghum root microbiome and enriches for monoderm bacteria. Proc Natl Acad Sci U S A.

[42] Bhattacharyya A, Pablo CHD, Mavrodi OV, Weller DM, Thomashow LS, Mavrodi DV. Rhizosphere plant-microbe interactions under water stress. In: Gadd GM, Sima S, editors. Advances in Applied Microbiology. Academic Press; 2021. pp. 65–113. 10.1016/bs.aambs.2021.03.001 PMC8959006 34140134

[43] Glick BR (2014). Bacteria with ACC deaminase can promote plant growth and help to feed the world. Microbiol Res.

[44] Gebauer L, Breitkreuz C, Heintz-Buschart A, Reitz T, Buscot F, Tarkka M, Bouffaud ML (2022). Water deficit history selects plant beneficial soil bacteria differently under conventional and organic farming. Front Microbiol.

[45] Jochum MD, McWilliams KL, Borrego EJ, Kolomiets MV, Niu G, Pierson EA, Jo YK (2019). Bioprospecting plant growth-promoting rhizobacteria that mitigate drought stress in grasses. Front Microbiol.

[46] Barnard RL, Osborne CA, Firestone MK (2013). Responses of soil bacterial and fungal communities to extreme desiccation and rewetting. ISME J.

[47] Furze JR, Martin AR, Nasielski J, Thevathasan NV, Gordon AM, Isaac ME (2017). Resistance and resilience of root fungal communities to water limitation in a temperate agroecosystem. Ecol Evol.

[48] Ochoa-Hueso R, Collins SL, Delgado-Baquerizo M, Hamonts K, Pockman WT, Sinsabaugh RL, Smith MD, Knapp AK, Power SA (2018). Drought consistently alters the composition of soil fungal and bacterial communities in grasslands from two continents. Glob Chang Biol.

[49] Bazany KE, Wang J-T, Delgado-Baquerizo M, Singh BK, Trivedi P (2022). Water deficit affects inter-kingdom microbial connections in plant rhizosphere. Environ Microbiol.

[50] Carbone MJ, Alaniz S, Mondino P, Gelabert M, Eichmeier A, Tekielska D, Bujanda R, Gramaje D (2021). Drought influences fungal community dynamics in the grapevine rhizosphere and root microbiome. J Fungi.

[51] Joergensen RG (1996). The fumigation-extraction method to estimate soil microbial biomass: calibration of the kec value. Soil Biol Biochem.

[52] Vance ED, Brookes PC, Jenkinson DS (1987). An extraction method for measuring soil microbial biomass C. Soil Eiol Biochem.

[53] Brookes PC, Landman A, Pruden G, Jenkinson DS (1985). Chloroform fumigation and the release of soil nitrogen: a rapid direct extraction method to measure microbial biomass nitrogen in soil. Soil Biol Biochem.

[54] ECPD. The european cultivated potato database. 2005. www.europotato.org. Accessed 2 Apr 2020.

[55] Griffiths RI, Whiteley AS, O’Donnell AG, Bailey MJ (2000). Rapid method for coextraction of DNA and RNA from natural environments for analysis of ribosomal DNA- and rRNA-based microbial community composition. Appl Environ Microbiol.

[56] Tedersoo L, Bahram M, Põlme S, Kõljalg U, Yorou NS, Wijesundera R, Ruiz LV, Vasco-Palacios AM, Thu PQ, Abarenkov K (2014). Global diversity and geography of soil fungi. Sci (80-).

[57] Parada AE, Needham DM, Fuhrman JA (2016). Every base matters: asssessing small subunit rRNA primers for marine microbiomes with mock communities, time series and global field samples. Environ Microbiol.

[58] Apprill A, Mcnally S, Parsons R, Weber L (2015). Minor revision to V4 region SSU rRNA 806R gene primer greatly increases detection of SAR11 bacterioplankton. Aquat Microb Ecol.

[59] Afgan E, Baker D, van den Beek M, Blankenberg D, Bouvier D, Čech M, Chilton J, Clements D, Coraor N, Eberhard C, Grüning B, Guerler A, Hillman-Jackson J, Kuster G, Von, Rasche E, Soranzo N, Turaga N, Taylor J, Nekrutenko A, Goecks J (2016). The galaxy platform for accessible, reproducible and collaborative biomedical analyses: 2016 update. Nucleic Acids Res.

[60] Martin M (2011). Cutadapt removes adapter sequences from high-throughput sequencing reads. EMBnet J.

[61] Andrews S, FastQC A. A quality control tool for high throughput sequence data. 2010. https://www.bioinformatics.babraham.ac.uk/projects/fastqc/http://www.bioinformatics.bbsrc.ac.uk/projects/fastqc/. Accessed 9 Jan 2023.

[62] Callahan BJ, McMurdie PJ, Rosen MJ, Han AW, Johnson AJA, Holmes SP (2016). DADA2: high-resolution sample inference from Illumina amplicon data. Nat Methods.

[63] Abarenkov K, Zirk A, Piirmann, Timo, Pöhönen R, Ivanov F, Nilsson R, Henrik. Kõljalg U. UNITE QIIME release for fungi. UNITE community. 2022. 10.15156/BIO/2483916. Accessed 16 Jan 2023.

[64] Davis NM, Proctor DM, Holmes SP, Relman DA, Callahan BJ (2018). Simple statistical identification and removal of contaminant sequences in marker-gene and metagenomics data. Microbiome.

[65] Khan MA, Gemenet DC, Villordon A (2016). Root system architecture and abiotic stress tolerance: current knowledge in root and tuber crops. Front Plant Sci.

[66] Zinta R, Tiwari JK, Buckseth T, Thakur K, Goutam U, Kumar D, Challam C, Bhatia N, Poonia AK, Naik S, Singh RK, Thakur AK, Dalamu D, Luthra SK, Kumar V, Kumar M (2022). Root system architecture for abiotic stress tolerance in potato: lessons from plants. Front Plant Sci.

[67] Comas LH, Becker SR, Cruz VMV, Byrne PF, Dierig DA (2013). Root traits contributing to plant productivity under drought. Front Plant Sci.

[68] Zarzyńska K, Boguszewska-Mańkowska D, Nosalewicz A (2017). Differences in size and architecture of the potato cultivars root system and their tolerance to drought stress. Plant Soil Environ.

[69] Boguszewska-Mańkowska D, Zarzyńska K, Nosalewicz A (2020). Drought differentially affects root system size and architecture of potato cultivars with differing drought tolerance. Am J Potato Res.

[70] Gervais T, Creelman A, Li XQ, Bizimungu B, De Koeyer D, Dahal K (2021). Potato response to drought stress: physiological and growth basis. Front Plant Sci.

[71] Albiski F, Najla S, Sanoubar R, Alkabani N, Murshed R (2012). In vitro screening of potato lines for drought tolerance. Physiol Mol Biol Plants.

[72] Pinheiro Alves de Souza Y, Schloter M, Weisser W, Schulz S (2023). Deterministic development of soil microbial communities in disturbed soils depends on microbial biomass of the bioinoculum. Microb Ecol.

[73] Ling N, Wang T, Kuzyakov Y (2022). Rhizosphere bacteriome structure and functions. Nat Commun.

[74] Sun X, Wang N, Li P, Jiang Z, Liu X, Wang M, Su Z, Zhang C, Lin F, Liang Y (2020). Endophytic fungus *Falciphora Oryzae* promotes lateral root growth by producing indole derivatives after sensing plant signals. Plant Cell Environ.

[75] Friedman M (2006). Potato glycoalkaloids and metabolites: roles in the plant and in the diet. J Agric Food Chem.

[76] Friedman M (2002). Tomato glycoalkaloids: role in the plant and in the diet. J Agric Food Chem.

[77] Milner SE, Brunton NP, Jones PW, Brien NMO, Collins SG, Maguire AR (2011). Bioactivities of glycoalkaloids and their aglycones from *Solanum* species. J Agric Food Chem.

[78] Nakayasu M, Takamatsu K, Yazaki K, Sugiyama A (2023). Plant specialized metabolites in the rhizosphere of tomatoes: secretion and effects on microorganisms. Biosci Biotechnol Biochem.

[79] Bulgarelli D, Schlaeppi K, Spaepen S, van Themaat EVL, Schulze-Lefert P (2013). Structure and functions of the bacterial microbiota of plants. Annu Rev Plant Biol.

[80] Dennis PG, Miller AJ, Hirsch PR (2010). Are root exudates more important than other sources of rhizodeposits in structuring rhizosphere bacterial communities?. FEMS Microbiol Ecol.

[81] Shi S, Richardson AE, O’Callaghan M, Deangelis KM, Jones EE, Stewart A, Firestone MK, Condron LM (2011). Effects of selected root exudate components on soil bacterial communities. FEMS Microbiol Ecol.

[82] De Vries FT, Griffiths RI, Knight CG, Nicolitch O, Williams A (2020). Harnessing rhizosphere microbiomes for drought-resilient crop production. Sci (80-).

[83] Micallef SA, Shiaris MP, Colón-Carmona A (2009). Influence of *Arabidopsis thaliana* accessions on rhizobacterial communities and natural variation in root exudates. J Exp Bot.

[84] Gschwendtner S, Esperschütz J, Buegger F, Reichmann M, Müller M, Munch JC, Schloter M (2011). Effects of genetically modified starch metabolism in potato plants on photosynthate fluxes into the rhizosphere and on microbial degraders of root exudates. FEMS Microbiol Ecol.

[85] Canarini A, Merchant A, Dijkstra FA (2016). Drought effects on *Helianthus annuus* and *Glycine max* metabolites: from phloem to root exudates. Rhizosphere.

[86] Preece C, Peñuelas J (2016). Rhizodeposition under drought and consequences for soil communities and ecosystem resilience. Plant Soil.

[87] Song F, Han X, Zhu X, Herbert SJ (2012). Response to water stress of soil enzymes and root exudates from drought and non-drought tolerant corn hybrids at different growth stages. Can J Soil Sci.

[88] Macias-Benitez S, Garcia-Martinez AM, Caballero Jimenez P, Gonzalez JM, Tejada Moral M, Parrado Rubio J (2020). Rhizospheric organic acids as biostimulants: monitoring feedbacks on soil microorganisms and biochemical properties. Front Plant Sci.

[89] Badri DV, Chaparro JM, Zhang R, Shen Q, Vivanco JM (2013). Application of natural blends of phytochemicals derived from the root exudates of *Arabidopsis* to the soil reveal that phenolic-related compounds predominantly modulate the soil microbiome. J Biol Chem.

[90] Faist H, Trognitz F, Antonielli L, Symanczik S, White PJ, Sessitsch A (2023). Potato root-associated microbiomes adapt to combined water and nutrient limitation and have a plant genotype-specific role for plant stress mitigation. Environ Microbiome.

[91] Huang YM, Zou YN, Wu QS (2017). Alleviation of drought stress by mycorrhizas is related to increased root H2O2 efflux in trifoliate orange. Sci Rep.

[92] Voothuluru P, Sharp RE (2013). Apoplastic hydrogen peroxide in the growth zone of the maize primary root under water stress. I. increased levels are specific to the apical region of growth maintenance. J Exp Bot.

[93] Lee EJ, Karoonuthaisiri N, Kim HS, Park JH, Cha CJ, Kao CM, Roe JH (2005). A master regulator σB governs osmotic and oxidative response as well as differentiation via a network of sigma factors in Streptomyces coelicolor. Mol Microbiol.

[94] Leirós M, Alonso E, Sanchez JA, Rateb ME, Ebel R, Houssen WE, Jaspars M, Alfonso A, Botana LM (2014). Mitigation of ROS insults by *Streptomyces* secondary metabolites in primary cortical neurons. ACS Chem Neurosci.

[95] Kopecky J, Samkova Z, Sarikhani E, Kyselková M, Omelka M, Kristufek V, Divis J, Grundmann GG, Moënne-Loccoz Y, Sagova-Mareckova M (2019). Bacterial, archaeal and micro-eukaryotic communities characterize a disease-suppressive or conducive soil and a cultivar resistant or susceptible to common scab. Sci Rep.

[96] Wei Q, Li J, Yang S, Wang W, Min F, Guo M, Zhang S, Dong X, Hu L, Li Z, Wang X (2022). *Streptomyces rhizophilus* causes potato common scab Disease. Plant Dis.

[97] Lugtenberg B, Kamilova F (2009). Plant-growth-promoting rhizobacteria. Annu Rev Microbiol.

[98] Vacheron J, Desbrosses G, Bouffaud ML, Touraine B, Moënne-Loccoz Y, Muller D, Legendre L, Wisniewski-Dyé F, Prigent-Combaret C (2013). Plant growth-promoting rhizobacteria and root system functioning. Front Plant Sci.

[99] Mattoo AK, Suttle JC (2017). The plant hormone Ethylene. 1st Editio.

[100] Tanimoto M, Roberts K, Dolan L (1995). Ethylene is a positive regulator of root hair development in *Arabidopsis thaliana*. Plant J.

[101] Gamalero E, Glick BR, Ahmad P, Prasad M (2012). Ethylene and abiotic stress tolerance in plants. Environmental adaptations and stress tolerance of plants in the era of climate change.

[102] Malicka M, Magurno F, Piotrowska-Seget Z (2022). Plant association with dark septate endophytes: when the going gets tough (and stressful), the tough fungi get going. Chemosphere.

[103] Lozano YM, Aguilar-Trigueros CA, Roy J, Rillig MC (2021). Drought induces shifts in soil fungal communities that can be linked to root traits across 24 plant species. New Phytol.

[104] Buscardo E, Souza RC, Meir P, Geml J, Schmidt SK, da Costa ACL, Nagy L (2021). Effects of natural and experimental drought on soil fungi and biogeochemistry in an Amazon rain forest. Commun Earth Environ.

[105] Siebielec S, Siebielec G, Klimkowicz-Pawlas A, Gałazka A, Grzadziel J, Stuczynski T (2020). Impact of water stress on microbial community and activity in sandy and loamy soils. Agronomy.

[106] Liotti RG, da Silva Figueiredo MI, da Silva GF, de Mendonça EAF, Soares MA (2018). Diversity of cultivable bacterial endophytes in *Paullinia cupana* and their potential for plant growth promotion and phytopathogen control. Microbiol Res.

[107] Banerjee S, Palit R, Sengupta C, Standing D (2010). Stress induced phosphate solubilization by *Arthrobacter* sp. and *Bacillus* sp. isolated from tomato rhizosphere. Aust J Crop Sci.

[108] Boukhatem ZF, Merabet C, Tsaki H (2022). Plant growth promoting Actinobacteria, the most promising candidates as bioinoculants?. Front Agron.

[109] Singh P, Singh R, Madhu GS, Singh VP (2023). Seed biopriming with *Trichoderma Harzianum* for growth promotion and drought tolerance in rice (Oryza Sativus). Agric Res.

