# Supplementary material for: Cultivar-specific dynamics: unravelling rhizosphere microbiome responses to water deficit stress in potato cultivars
Source: BMC Microbiol. 2023 Dec 1;23:377. doi: 10.1186/s12866-023-03120-4 (PMC10691024; doi:10.1186/s12866-023-03120-4)
Supplement: Supplementary file 1 — Supplementary Material 1 [file 12866_2023_3120_MOESM1_ESM.docx]

# Supplementary methods

**Table S1:** Standard protocols used for measurement of soil physico-chemical properties.

| **Measurements** | **Supplier** | **Reference** |
| --- | --- | --- |
| pH | AGROLAB Agrarzentrum GmbH | VDLUFA I, A5.1.1: 2016 |
| P_2_O_5_ | AGROLAB Agrarzentrum GmbH | VDLUFA I, A6.2.1.1: 2012 |
| K_2_O | AGROLAB Agrarzentrum GmbH | VDLUFA I, A6.2.1.1: 2012 |
| Mg | AGROLAB Agrarzentrum GmbH | VDLUFA I, A6.2.4.1: 1991 |
| Clay (<0.002 mm) | AGROLAB Agrarzentrum GmbH | DIN ISO 11277: 2002-08 |
| Silt (0.002 – 0.063 mm) | AGROLAB Agrarzentrum GmbH | DIN ISO 11277: 2002-08 |
| Sand (0.063 – 2 mm) | AGROLAB Agrarzentrum GmbH | DIN ISO 11277: 2002-08 |

## Sampling and measurement of plant growth parameters

On day 42, the experiment was terminated. The plants were carefully removed from pots and the rhizosphere soil samples were promptly collected and transferred in 2 ml Eppendorf tubes. To preserve the samples, the tubes were immediately placed on dry ice and subsequently stored in a freezer at -80°C until DNA extraction could be performed. Stem height and root length measurements were taken using a ruler. Fresh leaves from each plant were carefully collected and placed into petri dishes. The fresh weight of the leaves was then measured using a scale (Sartorius GmbH, Göttingen, Germany). Following this, the leaves were dried for 2 days in an oven set at 75°C and dry weight was promptly recorded.

One rhizosphere sample from the cultivar HERBST grown in DSM soil under reduced watering was excluded from the library preparation due to technical issues encountered during sampling. Following sequencing, two samples were excluded from fungal dataset due to a significantly low read count. As a result, there were four biological replicates for the cultivar MONI in DSM soil under reduced watering and four biological replicates in DSM source soil for subsequent analyses.

# Supplementary tables and figures

**Table S2:** Measurement of the soil microbial biomass (Cmic and Nmic), dissolved organic carbon (DOC), nitrogen (DON), inorganic nitrogen (NH_4_^+^, NO_3_^-^), soil texture (Sand, Silt, Clay), pH, and mineral contents (P_2_O_5_, K_2_O, Mg) in the bare NSM, DSM prior to planting. Mean values are reported in the table and statistical significance was calculated using either paired t-test or Wilcoxon test (p < 0.05, n = 3).

|  |  |  |  |  |  |  |  |  |
| --- | --- | --- | --- | --- | --- | --- | --- | --- |
|  |  | **Cmic** | **DOC** | **Nmic** | **DON** | **NH_4_^+^** | **NO_3_^-^** | **mWHC** |
|  |  | µg/g dry soil | µg/g dry soil | µg/g dry soil | µg/g dry soil | µg/g dry soil | µg/g dry soil | % |
|  | DSM | 94.08 | 279.61 | 9.64 | 30.36 | 7.26 | 2.4100 | 26.2400 |
|  | NSM | 159.06 | 26.33 | 20.86 | 7.1 | 0.16 | 5.1300 | 34.5800 |
|  | p-value | 0.004 | <2.2e-16 | 0.004 | <9.28e-14 | 0.0006 | 0.012 | 0.05 |
|  | Method | Student t-test | Student t-test | Wilcoxon test | Student t-test | Student t-test | Student t-test | Student t-test |
|  |  |  |  |  |  |  |  |  |
|  |  |  |  |  |  |  |  |  |
|  |  | **P_2_O_5_** | **K_2_O** | **Mg** | **pH** | **Sand** | **Silt** | **Clay** |
|  |  | mg/100g dry soil | mg/100g dry soil | µg/100g dry soil | - | % | % | % |
|  | DSM | 24.33 | 25.33 | 6.60 | 7.43 | 44 | 40 | 16 |
|  | NSM | 24 | 26 | 8.70 | 7.46 | 44 | 40 | 16 |
|  | p-value | 1 | 0.35 | 0.007 | 1 | 1 | 1 | - |
|  | Method | Wilcoxon test | Wilcoxon test | Student t-test | Wilcoxon test | Student t-test | Wilcoxon test |  |
|  |  |  |  |  |  |  |  |  |
|  |  |  |  |  |  |  |  |  |


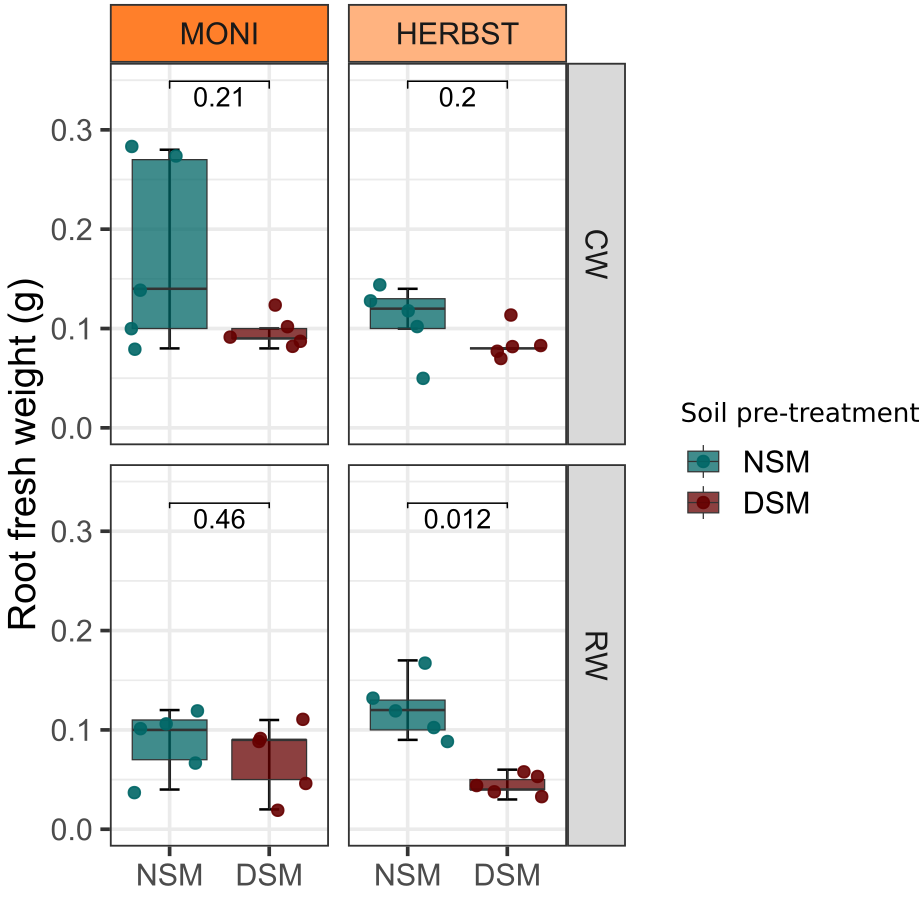


**Figure S1.** Plant growth parameters. Root fresh weight of two potato cultivars MONI and HERBST were compared between native soil with natural microbiome (NSM, turquoise green) and autoclaved soil with disturbed microbiome (DSM, brown) under continuous watering (CW) and reduced watering (RW). Horizontal bars within boxes are the median. The tops and bottoms of the boxes represent 75th and 25th quartiles, respectively. The two vertical lines outside the boxes represent the whiskers. The colored dots stand for the individual observations. A non-parametric Wilcoxon test (p < 0.05, n = 5) was applied to calculate significant differences across sample groups and numbers above the boxes indicate the statistical p-values.


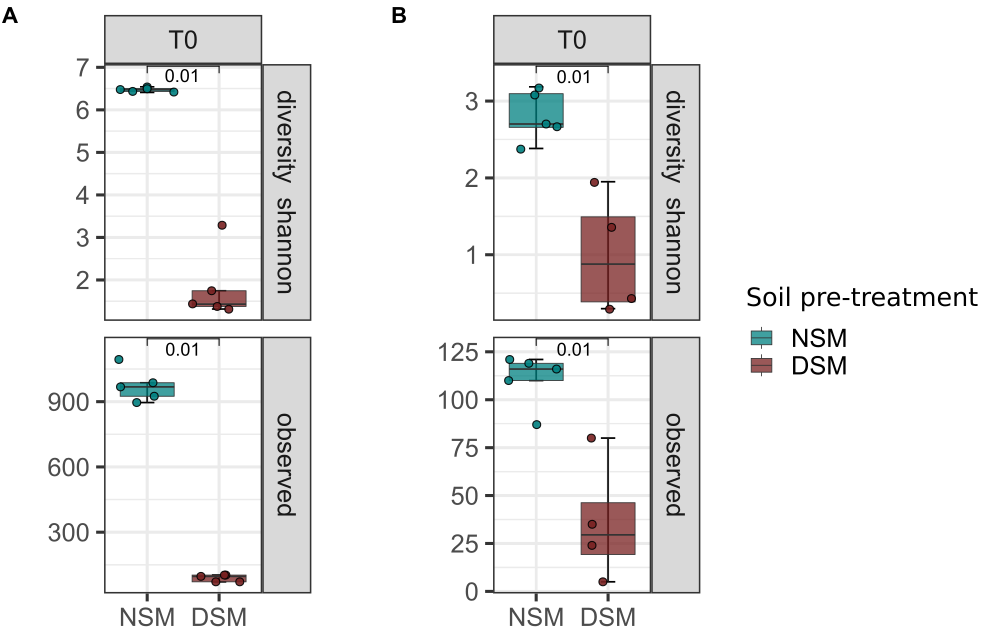


**Figure S2.** Microbial α-diversity in source soils. Shannon index and observed species of A) bacterial and B) fungal communities were compared between native soil with natural microbiome (NSM) and autoclaved soil with disturbed microbiome (DSM).  Boxplots display the medians, tops and bottoms of the boxes represent 75th and 25th quartiles, and whiskers outside this range; dots illustrate the individual observations in each sample group. A non-parametric Wilcoxon test (p < 0.05, n = 5) was applied to calculate significant differences across sample groups and numbers above the boxes indicate the corresponding p-values.


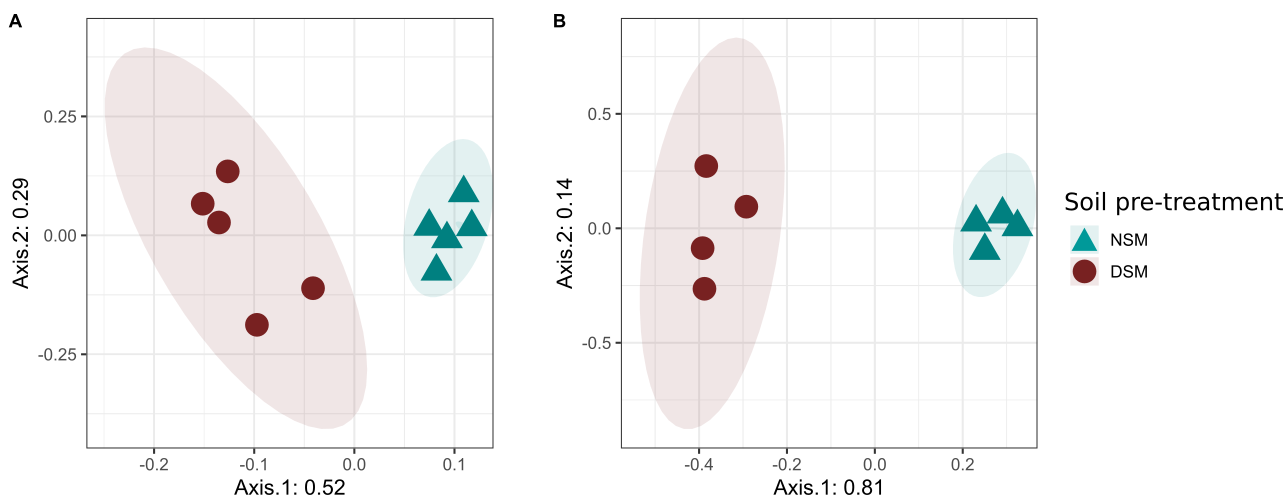


**Figure S3.** Ordination plots. Principal coordinate analysis (PCoA) of beta diversity in source soils for A) bacterial and B) fungal communities based on weighted UniFrac distance. Colored shapes represent soil pre-treatment (turquoise green triangles <= natural soil microbiome (NSM), and brown circles <= disturbed soil microbiome (DSM)). Soil pre-treatment was separated along the first axis.

**Figure S4.** Analysis of bacterial community composition. (A) Relative abundance of the top 9 classes in the rhizosphere of two potato cultivars MONI and HERBST cultivated in native soil with natural microbiome (NSM) and autoclaved soil with disturbed microbiome (DSM) under continuous watering (CW) and reduced watering (RW). Colors indicate the individual taxa at the class level.


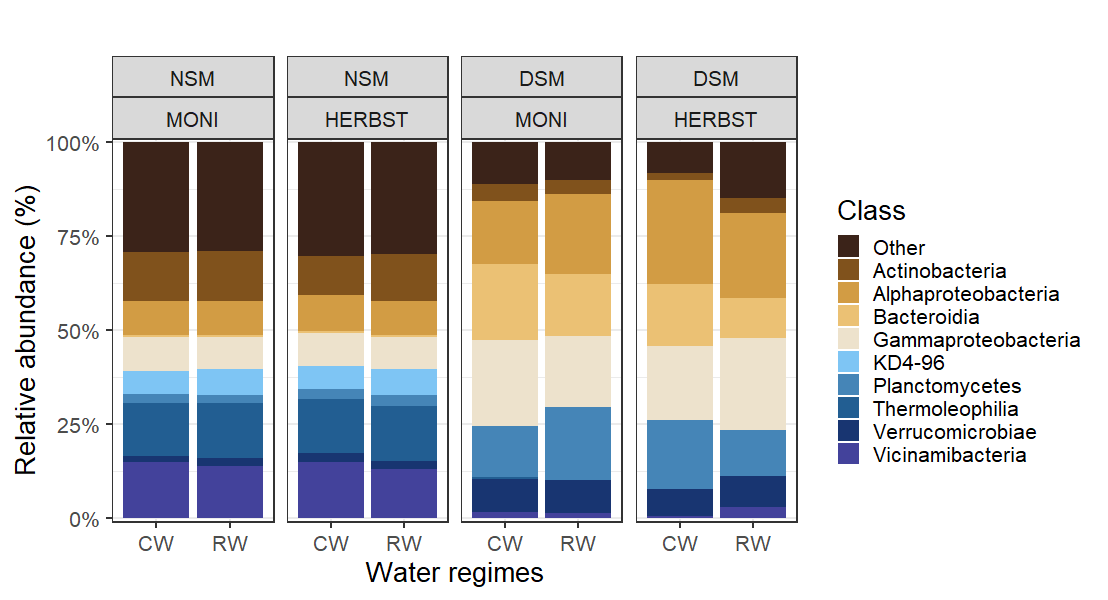


**Figure S5.** Analysis of bacterial community composition. (A) Relative abundance of the top 9 classes in the rhizosphere of two potato cultivars MONI and HERBST cultivated in native soil with natural microbiome (NSM) and autoclaved soil with disturbed microbiome (DSM) under continuous watering (CW) and reduced watering (RW). Colors indicate the individual taxa at the class level.


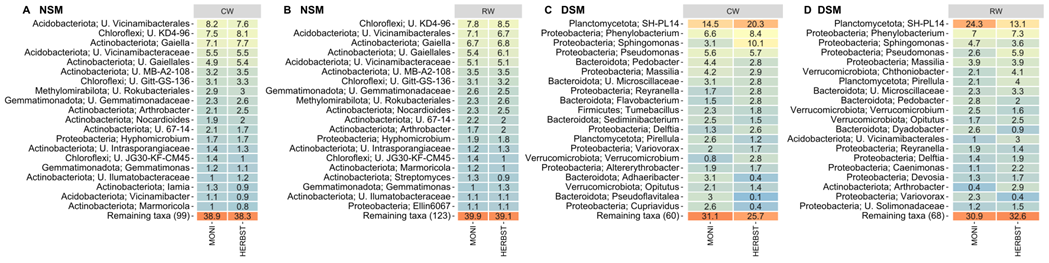
**Figure S6.**  Composition of shared bacterial community of two potato cultivars (MONI, HERBST) grown in natural (NSM) and disturbed (DSM) soil microbiomes under control (CW; A, C) and reduced watering (RW; B, D). Heatmap displays the top 20 taxa aggregated at the genus level. Numbers in the heatmap indicate the relative abundance of each taxon across sample groups.


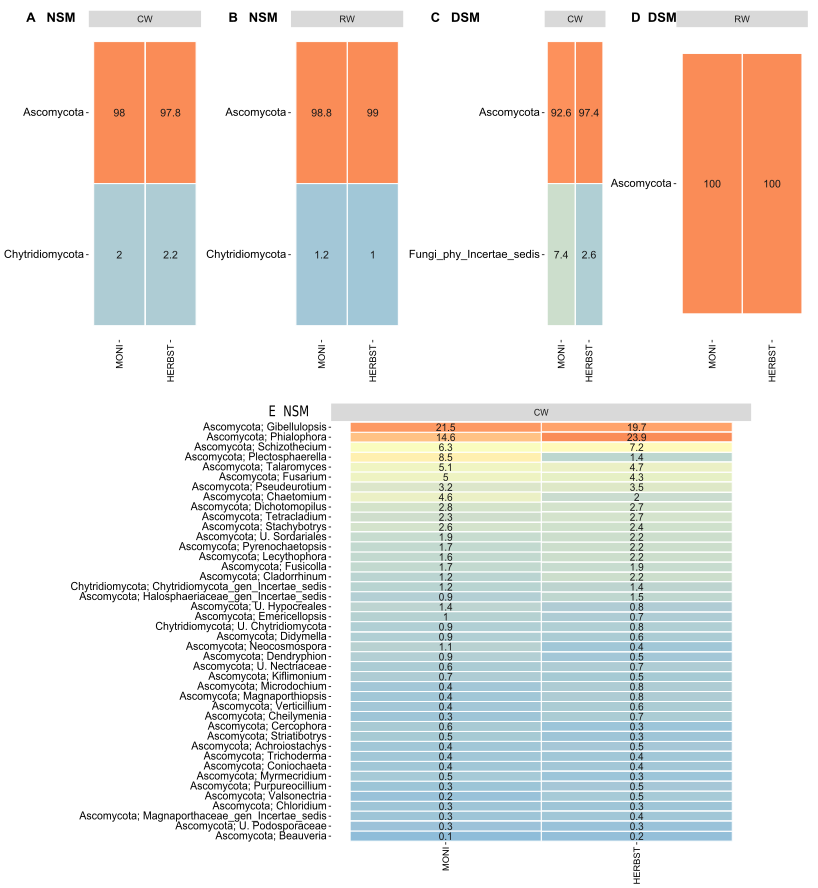
 **Figure S7.**  Composition of shared fungal community of two potato cultivars (MONI, HERBST) grown in natural (NSM) and disturbed (DSM) soil microbiomes under control (CW; A, C, E) and reduced watering (RW; B, D). Heatmap displays the top taxa aggregated at the phylum (A, B, C, D) and genus level. Numbers in the heatmap indicate the relative abundance of each taxon across sample groups.
